# Antarctic snow algae: unraveling the processes underlying microbial community assembly during blooms formation

**DOI:** 10.1186/s40168-023-01643-6

**Published:** 2023-09-05

**Authors:** Daniela F. Soto, Iván Gómez, Pirjo Huovinen

**Affiliations:** 1 Instituto de Ciencias Marinas y Limnológicas, Facultad de Ciencias, Campus Isla Teja, Universidad Austral de Chile, Valdivia, Chile; 2 Research Centre on Dynamics of High Latitude Marine Ecosystems (IDEAL), Valdivia, Chile

## Abstract

**Background and aims:**

At the West Antarctic Peninsula, snow algae blooms are composed of complex microbial communities dominated by green microalgae and bacteria. During their progression, the assembly of these microbial communities occurs under harsh environmental conditions and variable nutrient content due to fast snow melting. To date, it is still unclear what are the ecological mechanisms governing the composition and abundance of microorganisms during the formation of snow algae blooms. In this study, we aim to examine the main ecological mechanisms governing the assembly of snow algae blooms from early stages to colorful stages blooms.

**Methods:**

The composition of the microbial communities within snow algae blooms was recorded in the West Antarctic Peninsula (Isabel Riquelme Islet) during a 35-day period using 16S rRNA and 18S rRNA metabarcoding. In addition, the contribution of different ecological processes to the assembly of the microbial community was quantified using phylogenetic bin-based null model analysis.

**Results:**

Our results showed that alpha diversity indices of the eukaryotic communities displayed a higher variation during the formation of the algae bloom compared with the bacterial community. Additionally, in a macronutrients rich environment, the content of nitrate, ammonium, phosphate, and organic carbon did not play a major role in structuring the community. The quantification of ecological processes showed that the bacterial community assembly was governed by selective processes such as homogenous selection. In contrast, stochastic processes such as dispersal limitation and drift, and to a lesser extent, homogenous selection, regulate the eukaryotic community.

**Conclusions:**

Overall, our study highlights the differences in the microbial assembly between bacteria and eukaryotes in snow algae blooms and proposes a model to integrate both assembly processes.

Video Abstract

**Supplementary Information:**

The online version contains supplementary material available at 10.1186/s40168-023-01643-6.

## Introduction

The Maritime Antarctic has been regarded as one of the most sensitive world regions to global warming, with an accelerated ice and snow melting in the last decades [32, 40, 69]. This scenario has caused strong variations in the physicochemical characteristics of the cryosphere [26], providing favorable conditions for snow algae proliferation [20, 46]. However, climate change might also impact the processes such as dispersal phenology, successional trajectories [38], and the assembly of the microbial community of snow algae blooms. It has been reported that snow algae blooms might reduce the albedo by up to 20% at Maritime Antarctic snowfields [28]. Thus, the presence of snow algae does not only contribute to the productivity and biodiversity of the cryosphere, but also affects the energy balance at a global scale. However, despite the relevance of snow algae blooms, the processes governing the formation of these communities in their early stages are not well understood.

Although multiple mechanisms (e.g., selection, dispersal, drift, and speciation) may be responsible for controlling the diversity, distribution, and succession of the microbial community, quantifying their relative importance remains challenging [45]. Moreover, in the case of Antarctic snow ecosystems, logistical and temporal restrictions limit the study of these communities in their early stages. In the West Antarctic Peninsula, snow algae blooms are rapidly formed during the short spring period, which makes tracking the transition from fresh snow to colored snow algae blooms difficult.

Based on studies of visible and colorful snow algae blooms, it has been established that their functional profiles consistently exhibit genes involved in the tolerance to environmental stressors, which filter and select only adapted organisms [64]. Likewise, nutrient stoichiometry also affects the microbial community structure of snow algae blooms. For instance, the proliferation of snow algae is favored by the release of elements such as Fe, Mn, and P from melting glaciers [19], and abundances of snow algae species such as *Chloromonas cf. alpina* and *Chloromonas polyptera* have been shown to correlate positively with dissolved organic carbon (DOC) in the Arctic [36].

In addition, metagenomes of Antarctic snow algae blooms have shown that community functional potential was conserved among three different sites, regardless of the color of the bloom. The only significant differences were detected in genes related with tolerance to abiotic stress which were more abundant in one snow sample collected at Isabel Riquelme Islet [64]. These findings suggest that stochastic processes could explain partially the assembly of microbial communities in snow algae blooms [64].

Furthermore, genome-scale metabolic model reconstruction revealed potential biotic interactions within the bacterial community of snow algae blooms, e.g., competition for available resources. This could indicate that these communities show a low potential to self-support their nutrient requirements only the exchange of metabolites [64]. However, it has been reported that in the microbiome of some alpine snow algae communities, Proteobacteria like Oxalobacteraceae, Pseudomonadaceae, and Sphingomonadaceae show genus-specific co-occurrences with distinct species of microalgae [34]. Thus, as some co-cultivation experiments suggest, inter-kingdom mutualistic relationships are likely to occur in these systems [30, 34, 68].

Considering that the assembly of a community is a dynamic process, the mechanisms and composition of organisms may change over time [21]. In the present study, we examine the main ecological mechanisms operating during the early stages of the assembly of the microbial community within an Antarctic snowpack. In a 35-day field experiment, we collected successively snow samples from a locality in the West Antarctic Peninsula and witnessed the transition from white snow to colorful snow algae blooms during the spring–summer season. Using 16S rRNA and 18S rRNA gene metabarcoding, the temporal patterns in microbial diversity and some key functional interactions during the formation of snow algae blooms were assessed. We hypothesize that the formation of colorful snow algae blooms is mostly driven by selective ecological mechanisms and to a lesser extent by stochastic processes. Thus, a decline in the microbial diversity is expected during the formation of algal blooms because species that are not able to cope with environmental harsh conditions are filtered out. Likewise, cooperative interactions between algae and bacteria may be selecting and facilitating the colonization of the dominant species in snow algae blooms. Therefore, co-occurring patterns between bacteria and algae species are expected during the formation of snow algae bloom.

## Materials and methods

### Study sites

**Fig. 1:**
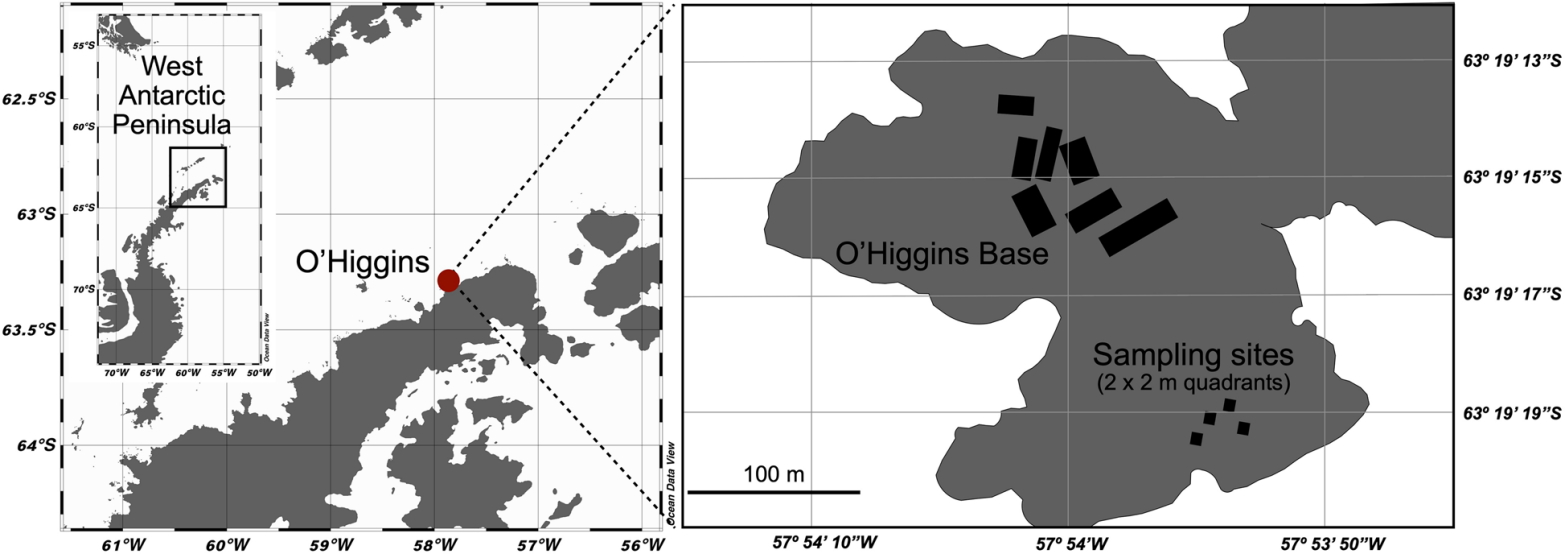
Location of the sampling site near the O’Higgins Base Station at the West Antarctic Peninsula. For full details, see Supplementary Table 1

Snow samples were collected from an area near the Chilean O’Higgins Base Station located on the Isabel Riquelme Islet (63° S; 57° W) during November and December of 2019 (Fig. 1). Snow from four 2 × 2 m quadrats was sampled twice a week over a 35-day period with a total of 46 samples.

The main criteria to choose the snow patches were that they should be in an area without constant human transit and with safe access to the sampling sites even during bad weather conditions. Between the first and second sampling days, the snow of the two sites melted completely; therefore, the quadrats were moved to other snow patches that fulfilled the previously stated criteria (see new coordinates in Supplementary Table 1). These two samples were not considered for further analyses. Moreover, because the progression of the snow algae blooms was unpredictable, two very light-green snow patches (sites 1 and 2) and two white snow patches (sites 3 and 4) were originally selected. The colors of the blooms changed during the experiment.

### Snow sample collection and preparation

For the molecular analyses (metabarcoding), superficial snow was sampled into two 1260-mL Whirl-Pak bags (Nasco, WI, USA) using sterile gloves and a small metallic shovel. In this study, each individual 2 × 2 m quadrant was considered a sampling point, thus during the time series, we swept the area randomly until the sampling bags were full, without digging deep into the snow patches.

After sampling, the snow was melted at a low temperature inside a cool box. Then, samples were filtered through 0.22-μm mixed cellulose esters membranes (47 mm, Millipore). Depending on the intensity of the bloom color, different volumes were filtered. For clear white snow, 1 L was filtered, while for colored snow, only 100–150 mL was filterable before the filter saturation. Finally, the filters were stored at −20 °C inside individual aluminum foil envelopes and transported in a dry shipper to the laboratory at the Universidad Austral de Chile (Valdivia). Here, samples were stored at −80 °C until further DNA extraction.

### Environmental parameters

For the determination of the nutrient content (NH_4_^+^, NO_3_^−^, and PO_4_^3−^), superficial snow samples were collected in a 1260-mL Whirl-Pak bag from each sampling point site, every time we sampled the snow patches. Additionally, for the determination of the content DOC, snow samples were collected using sterile 50-mL centrifuge tubes. After collection, snow samples were transported to the laboratory at O’Higgins Base Station for further processing.

The nutrient concentrations of NH_4_^+^, NO_3_^−^, and PO_4_^3−^ in snow samples were quantified using a portable photometer (9300 Kit, YSI Inc., Yellow Springs, OH, USA). This instrument measures the change of color when chemical reagents react with the sample and whose intensity is proportional to the concentration of the parameter under test. Previously, samples were melted and filtered through 0.45-µm mixed cellulose esters membranes (47 mm, Millipore). Afterwards, the filtered liquid was added to the commercial kit used for quantifying the nutrients following the manufacturer’s instructions. All samples were measured within 24 h after collection. For the determination of DOC, samples were stored at −80 °C and transported in a dry shipper to the Centro de Investigación en Ecosistemas de la Patagonia (CIEP) in Coyhaique for quantification using the combustion catalytic oxidation/non-dispersive infrared (NDIR) method [67]. The chlorophyll *a* concentration was determined in 5-mL aliquots of melted snow filtered onto Whatman GFF filters. The filters were kept frozen at −20 °C and transported to the Universidad Austral de Chile in Valdivia, until extraction in 90% ethanol at 75 °C, and spectrophotometric measurements [63]. No acid correction for phaeophytin was made. Daily records of maximum and minimum temperatures and snowfall at O’Higgins Base Station during the study period were obtained from the Dirección Meteorológica de Chile (https://climatologia.meteochile.gob.cl/).

### DNA extraction and metabarcoding

Total DNA from snow samples was extracted using the DNeasy PowerSoil Pro Kit (Qiagen, Hilden, Germany) following the instructions from the manufacturer. DNA was extracted from filtered samples thawed in ice and cut into small pieces with a sterile scalpel. Half of the filters were used for DNA extraction and the other half was kept at −20 °C as a backup. Negative controls using ultrapure water were included during the DNA extraction (one negative control for each 23 samples). Finally, DNA quantification was performed using the Qubit® dsDNA HS Assay Kit and a Qubit 3.0 fluorometer (Thermo Fisher Sci., Waltham, MA, USA). Samples were normalized to a DNA concentration of 5 ng/μL.

Metabarcoding libraries of the 16S rRNA and 18S rRNA genes were generated at the AUSTRAL-omics core research facility of the Universidad Austral de Chile. Primers 341F/805R were used for the amplification of the hypervariable regions V3–V4 of the 16S rRNA gene [33], while primers 528F/706R were used for the hypervariable region V4 of the 18S rRNA gene [10]. Both primer pairs included an Illumina-sequencing adapter, an index sequence, and a heterogeneity spacer according to the design of Fadrosh et al. [17]. For multiplexing, three pools (with 16, 18, and 18 samples each) of equimolar DNA concentration of 16S rRNA amplicons were generated. Each pool included two negative controls, one of them consisting of an indexed PCR product using ultrapure water as a template and the other one a negative control obtained during the DNA extraction. For the 18S rRNA amplicons, three pools (27, 20, and 5 samples per pool) were generated. Two negative controls were included in each pool. Finally, the protocol for the library preparation was the same as previously detailed in Soto et al. [64].

In the case of bioinformatic analyses of 16S rRNA and 18S rRNA gene amplicons, first samples were demultiplexed and barcodes were removed using an in-house python script. Demultiplexed raw sequences were analyzed using the open-source software package DADA2 v1.26.0 [9]. The pipeline consisted of filtering and trimming the raw sequences, setting the error rate of the amplicons datasets, inferring the number of true sequence variants, and obtaining the full denoised sequences. Finally, the taxonomy of the amplicons sequence variants (ASVs) was assigned using the SILVA v132 training set of reference sequences [55] for the 16S rRNA gene sequences and the PR2 v4.12.0 training set [22] for the 18S rRNA gene sequences. The taxonomy of Chlorophyta algae was manually curated blasting the sequences of each ASV against the Nucleotide collection nr/nt database (Genbank database). ASVs with the same best hit were classified into taxonomic groups named after the most representative taxon. The detailed list of the groups formed and the ASVs gathered in each group is provided in Supplementary Table 2. After filtering and chimera’s removal, a total of 1,536,943 unique sequences for the 16S rRNA gene amplicon were obtained, and 2450 ASVs were identified after removing singletons, sequences that occurred in only one sample, and potential contaminants. With regard to the 18S rRNA gene amplicon, we obtained a total of 3,511,719 unique sequences, and 500 ASVs were identified. Rarefaction curves were constructed to verify the sequencing depths for the samples (Supplementary Fig. 1).

### Inferring community assembly mechanisms

The sequence alignments of the 16S rRNA and 18S rRNA gene ASVs were conducted under the ClustalW method using the R package *msa* [5] with the default parameters. In addition, maximum likelihood phylogenetic trees were constructed using FastTree 2.1.11 [51] and the substitution model GTR+Γ [73].

Quantification of the relative influences of community assembly processes was assessed using the R package iCAMP, which is a phylogenetic bin-based null model framework [45]. iCAMP divided taxa into different groups (bins) based on their phylogenetic relationships. In this study, the generated bins were based on the phylogenetic relationships of all the ASVs within the microbial community. Then, the process governing each bin is identified based on null model analysis of the phylogenetic diversity using the beta Net Relatedness Index (βNRI) and taxonomic β-diversities with the modified Raup–Crick metric (RC). For each bin, the fraction of pairwise comparisons with βNRI < − 1.96 and βNRI > + 1.96 are considered as the percentages of homogeneous and heterogeneous selection, respectively [66]. Homogeneous selection is a deterministic process, leading to similar community structures caused by similar environmental conditions, while heterogeneous selection is a process that leads to dissimilar community structures because of heterogeneous environmental conditions.

Additionally, pairwise comparisons with |βNRI|≤ 1.96 and RC < − 0.95 are treated as the percentages of homogenizing dispersal, while those with RC > + 0.95 as dispersal limitation [66]. The remainders with |βNRI|≤ 1.96 and |RC|≤ 0.95 represent the percentages of drift, diversification, weak selection, and/or weak dispersal [45, 74]. Finally, to estimate the relative importance of individual processes at the whole community level, the fractions of individual processes across all bins are weighted by the relative abundance of each bin.

### Statistical analysis

Rarefaction curves were generated using the Vegan package under R v3.6.1 [47]. In addition, the Shannon-Wiener index and Chao 1 richness were calculated and illustrated using the packages phyloseq [42] and ggplot2 [71]. Metabarcoding RDA was based on Hellinger distance [35] which was calculated using the R package BiodiversityR [31] and the reads count of bacterial and eukaryotic ASVs. RDA models were constructed considering the concentration of NH_4_^+^, NO_3_^−^, PO_4_^3−^, and DOC as constraining variables and test the significance of each variable performing an ANOVA-like permutation test for redundancy analysis (permutations = 999).

Correlation (Pearson correlation) matrices between bacteria and eukaryote abundances were computed and plotted using the Hmisc and corrplot R packages, respectively [23, 70]. For this analysis, we used the bacterial and eukaryotic bins that most contributed to the assembly of the microbial communities and were taxonomically assigned at their most resolved level. Finally, the figures were generated using the R package ggplot2.

## Results

### Temporal variation on the microbial composition of snow algae blooms

**Fig. 2:**
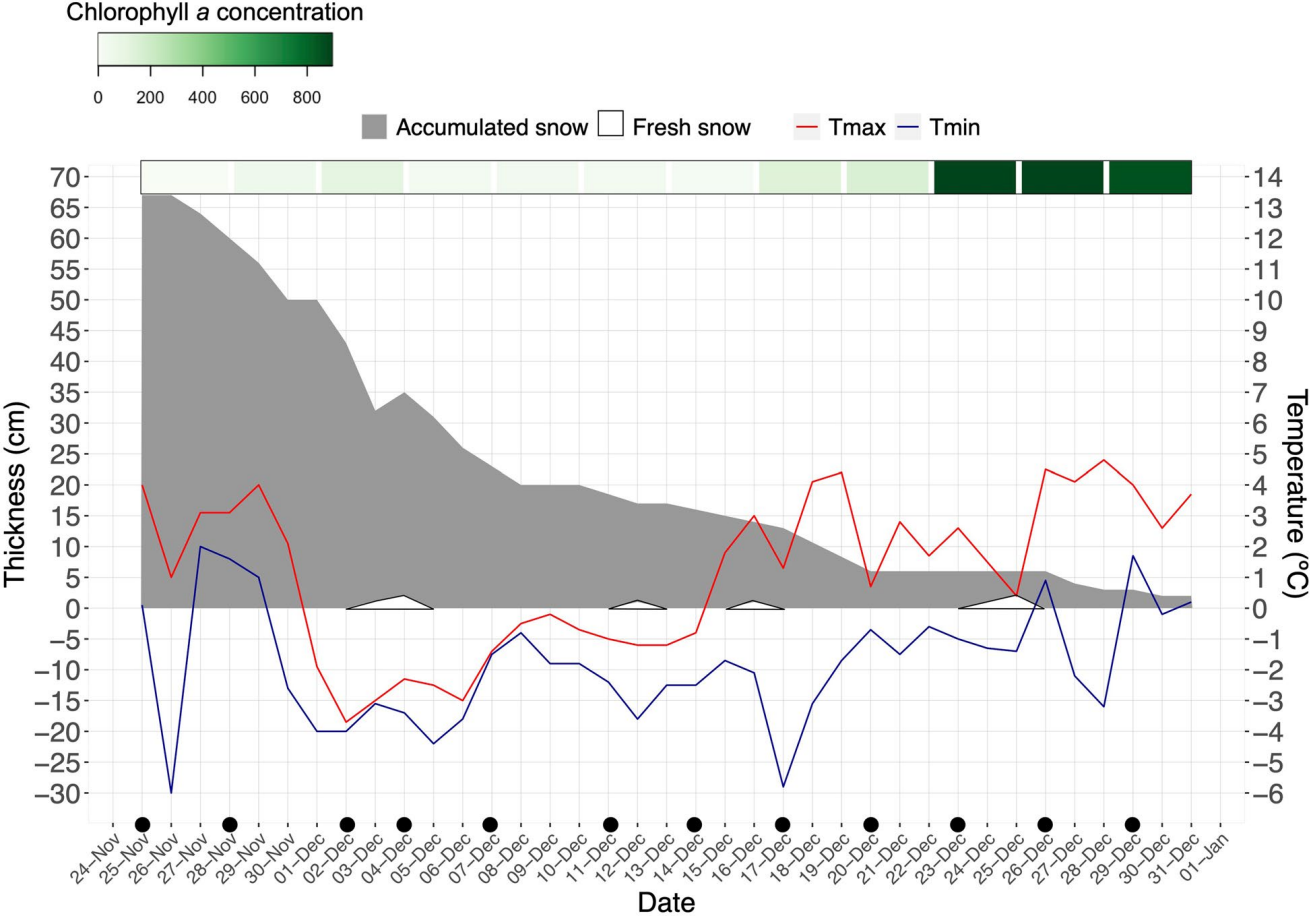
Snow conditions and air temperature during the time series at O’Higgins Base Station. Accumulated snow, fresh snow, and minimum and maximum atmospheric temperatures were recorded from November 25, 2019, to December 31, 2019. Black dots indicate the sampling days, and the green bar at the top illustrates the Chl *a* (μg/L) and the average concentration in four snow packs on the same sampling dates

**Fig. 3:**
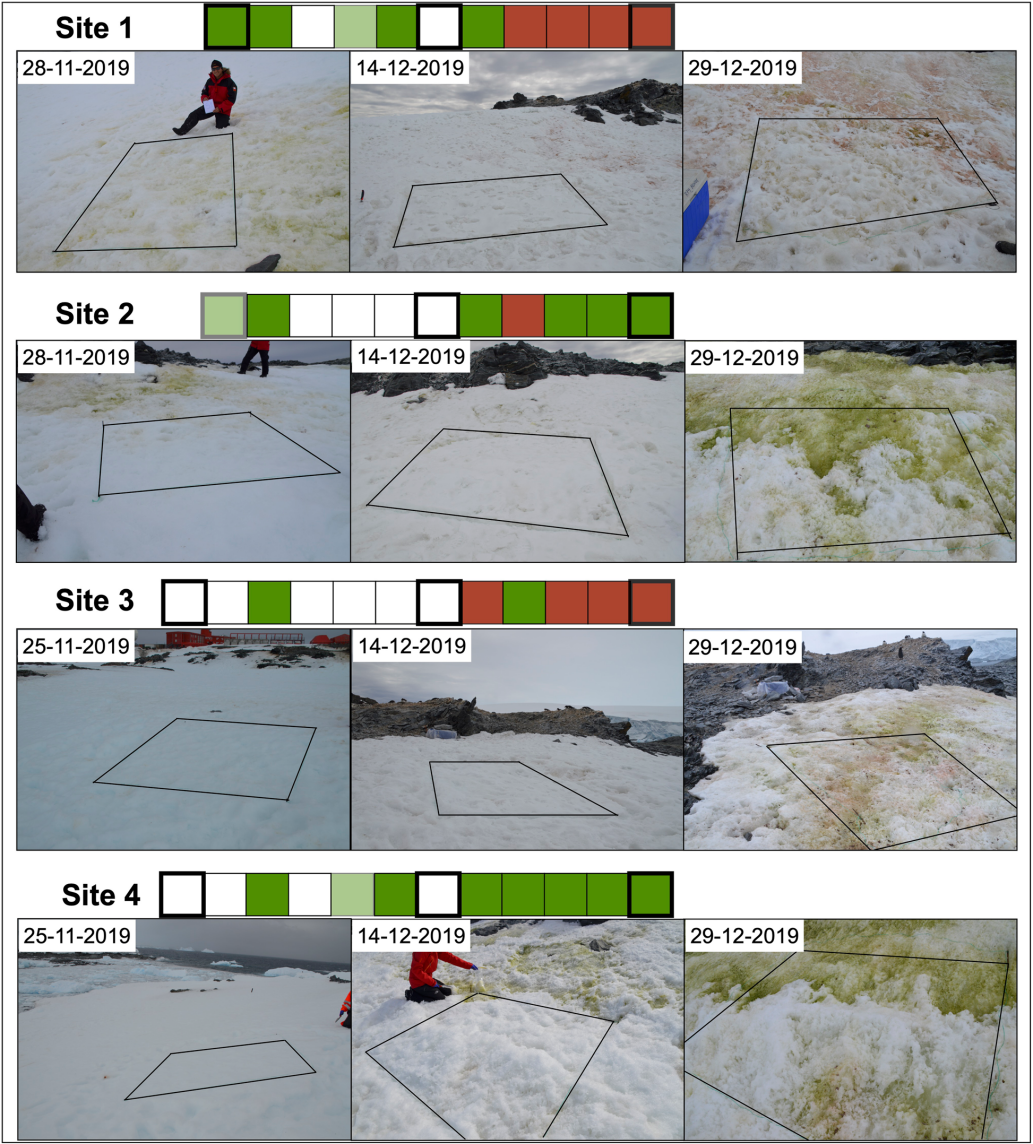
Time series of snow algal bloom development. The coordinates of each sampling site (2 × 2 m quadrants) are indicated in Supplementary Table 1. Snow color on each sampling day is indicated in the bar above each figure. Referential photos (date shown in the top left corner) illustrate the sampling days highlighted by bold borders squares

During the field experiment, over 60 cm of accumulated snow melted over a 35-day period (Fig. 2). In addition, snowing led to the accumulation of 2–3 cm of fresh snow, which melted fast due to the increase in air temperatures above 0 °C during the second half of December (Fig. 2). This increase was also associated with the development of the snow algae blooms, reflected in an increase of Chl *a* towards the end December (Fig. 2, Table 1), producing both green and red blooms (Fig. 3).

**Table 1:** Mean values of the chlorophyll a concentration in snow samples at each sampling day. Chl a concentration of every sample is detailed in Supplementary Table 1

**Date**	***Chl a (μg/L)***	
	*Mean*	*sd*
*25-11-19*	2.38	± 2.02
*28-11-19*	47.26	± 79.83
*02-12-19*	108.59	± 57.51
*04-12-19*	20.13	± 13.43
*07-12-19*	37.69	± 38.66
*11-12-19*	26.97	± 24.94
*14-12-19*	15.07	± 13.24
*17-12-19*	142.80	± 101.44
*20-12-19*	156.19	± 50.11
*23-12-19*	895.97	± 524.19
*26-12-19*	892.50	± 991.68
*29-12-19*	859.78	± 733.20

**Fig. 4:**
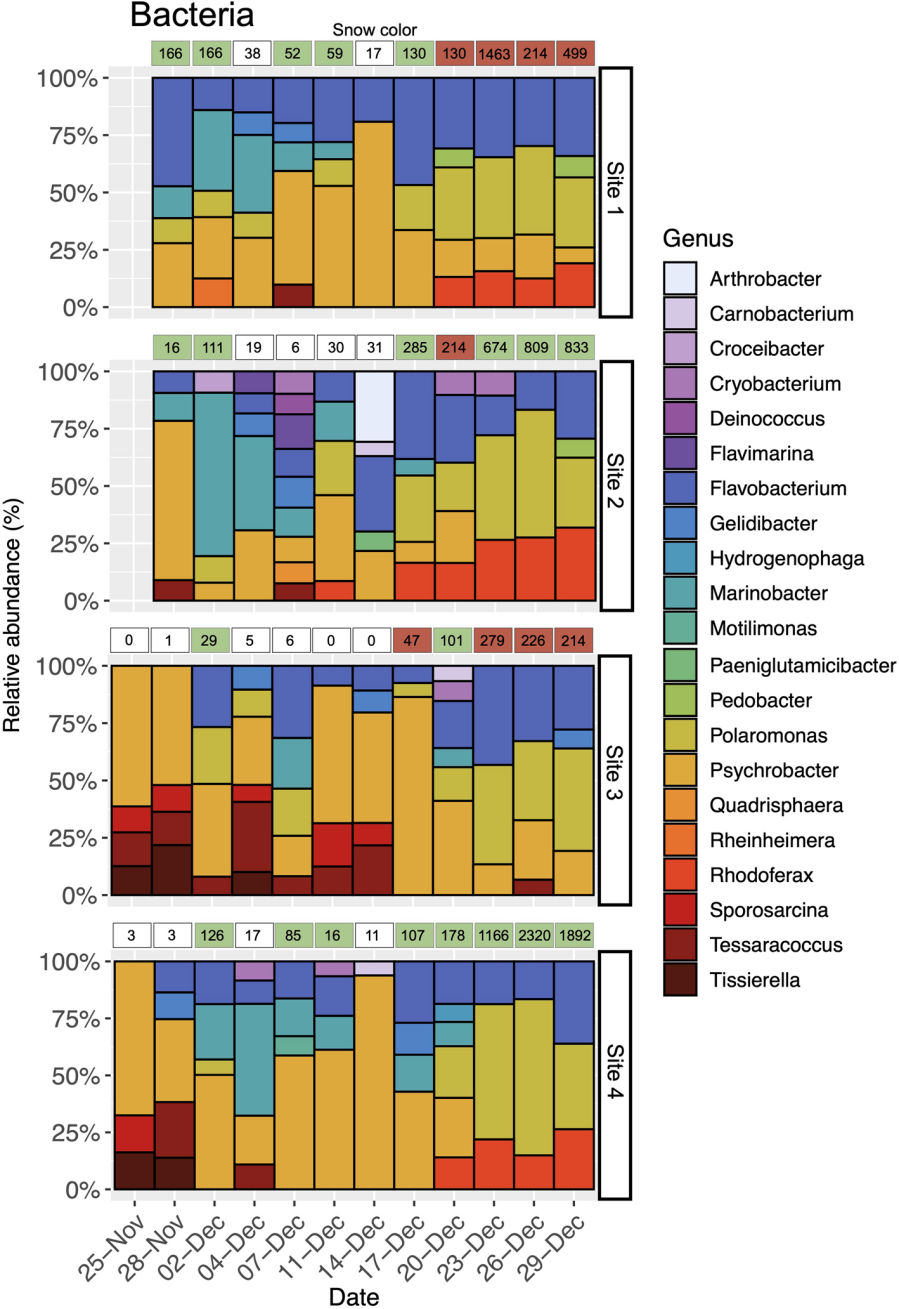
Changes in the bacteria composition within snow algae blooms. For illustrative purposes, only genera with mean relative abundances higher than 5% were considered. Snow colors as in Fig. 3. Chlorophyll *a* concentrations (μg/L) are shown inside the boxes that illustrate the color of the blooms at each sampling date

At the beginning of the temporal series (November 25), we observed that sites 3 and 4 displayed a similar bacterial composition, except for the bacterial genus *Tessaracoccus* that only was present at site 3, reaching a relative abundance of 12%. In these two sites, the bacterial genus *Psychrobacter* was the dominant taxon, accounting for more than 60% (relative abundance) of the most abundant sequences. Likewise, the bacterial genus *Tissierella* reached similar relative abundances in sites 3 and 4 (13% and 16%, respectively), while the genus *Sporosarcina* reached 11% and 16%, respectively (Fig. 4). As the snow algae blooms progressed, the composition of the bacterial communities changed. In the case of site 3, we observed a fluctuating relative abundance of *Flavobacterium*, *Polaromonas*, and *Tessaracoccus*. Also, we recorded a decline in the relative abundance of *Psychrobacter* towards the end of the field experiment (December 29) down to 19% of the most abundant sequences in the bacterial community (Fig. 4). In addition, the bacterial composition in site 4 was characterized by the changing relative abundances of *Marinobacter*, *Flavobacterium*, and *Psychrobacter*. In contrast with the observations on site 3, the relative abundance of *Psychrobacter* bacteria was so low at the end of the field experiment, where it did not reach 5% and thus did not appear in Fig. 4. However, despite the differences observed during the progression of the bloom, on December 29, sites 3 and 4 overall showed a high relative abundance of bacteria of the genera *Flavobacterium* and *Polaromonas*. Site 4 though also was characterized by the presence of *Rhodoferax* bacteria that reached up to a relative abundance of 26% (Fig. 4).

In the case of sites 1 and 2, the progression of the bloom was recorded from November 29. At first, the bacterial community at site 1 was dominated by *Flavobacterium* (47%), *Psychrobacter* (28%), *Marinobacter* (14%), and *Polaromonas* (11%). In contrast, site 2 was mostly dominated by *Psychrobacter* bacteria whose relative abundance peaks up to 69%, while *Flavobacterium*, *Marinobacter*, and *Tessaracoccus* reached relative abundances around 10% (Fig. 4). Similar to what was observed in site 4, the relative abundance of *Marinobacter* fluctuated during the bloom progression. In addition, at the end of the field survey (December 29), sites 1 and 2 were dominated by bacteria of the genera *Flavobacterium*, *Polaromonas*, and *Rhodoferax*, similar to what was observed at sites 3 and 4 (Fig. 4).

**Fig. 5:**
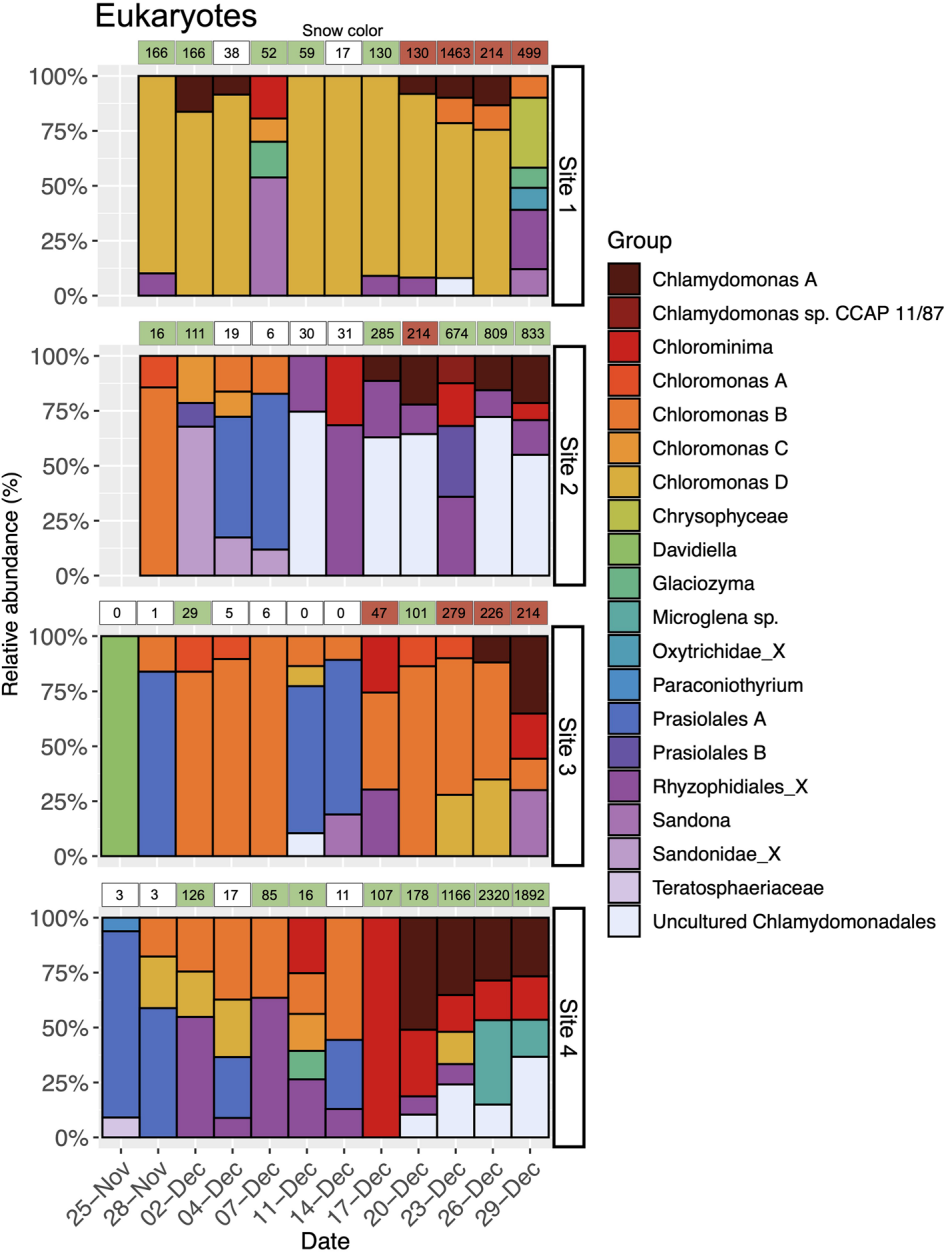
Changes in the eukaryotes composition within snow algae blooms. For illustrative purposes, only genera with mean relative abundances higher than 5% were considered. Snow colors as in Fig. 3. Chlorophyll *a* concentrations (μg/L) are shown inside the boxes that illustrate the color of the blooms at each sampling date

Regarding the eukaryotic community, the algae blooms of the four study sites displayed different progressions of their community structure. On November 25, the bloom on site 3 was dominated by *Davidiella* fungi. This taxon was the only one that reached a relative abundance higher than 5% and thus is the only one that appears in Fig. 5. In contrast, the bloom on site 4 was dominated by taxa classified into the group *Prasiolales* A algae that accounted for 85% of the reads (Fig. 5). The progression of blooms at site 3 showed a clear dominance of *Chloromonas* (group *Chloromonas* B) that turned red towards the end of the field experiment. In contrast, the bloom on site 4 was dominated by a mix of algal species such as *Chlorominima*, *Chlamydonomas* A, and *Microglena* sp. groups. In addition, this was a green bloom that never turned red during the field experiment (Fig. 5). Blooms on sites 1 and 2 were recorded from November 29 and showed that the first site was dominated by algae of the group *Chloromonas* D whose relative abundance accounts by 90% of the most abundant taxa, and the latter was dominated by algae belonging to the group *Chloromonas* B that reached up to the 85% of the reads included in Fig. 5. Thus, these blooms likely started earlier than those at sites 3 and 4. Towards the end of the field experiment, the bloom in site 1 was still dominated by *Chloromonas* D algae, but on December 29, the composition of the blooms was a mix of fungal and algal species. Also, this bloom was one of those that turned red towards the end of the study (Fig. 5). Finally, the bloom at site 2 progressed with increasing abundances of algae that could not be classified taxonomically at a deep level: here, uncultured Chlamydomonadales reached a relative abundance of 55% on December 29. In addition, a fluctuating, but abundant presence of Rhyzophidiales fungi was also recorded in this bloom reaching relative abundances between 12 and 68% within the most abundant taxa (Fig. 5).

### Diversity and richness of snow algae blooms

Results of the Shannon-Wiener index and Chao1 richness index (Supplementary Fig. 2) indicated that, in general, eukaryotic communities were less diverse and rich than bacterial communities but displayed a higher variation during the formation of the algae bloom. The bacterial communities on sites 1 and 3 were similar in terms of species diversity and richness during the formation of the blooms. In both sites, the Shannon-Wiener index reached a mean value of 5.8 ± 0.3 (mean ± sd). In the case of the Chao1 index, it reached a mean value of 629 ± 187 on site 3 and 606 ± 182 on site 1. Likewise, sites 4 and 2 were similar to each other reaching a Shannon-Wiener index mean value of 5.2 ± 0.3, while the Chao1 values were 413 ± 197 for site 2 and 434 ± 271 for site 4.

In the case of eukaryotes, the four sites showed a different pattern. The highest average diversity in terms of the Shannon-Wiener index was recorded on site 4 with a mean value of 2.7 ± 0.5 and the lowest on site 1 with a mean value of 2.0 ± 0.6. Likewise, in terms of species richness, the highest Chao1 score was recorded on site 4 with a mean value of 84 ± 44, and the lowest value was 55 ± 22 recorded at site 1.

### Influence of nutrients content in the microbial community structure

**Fig. 6:**
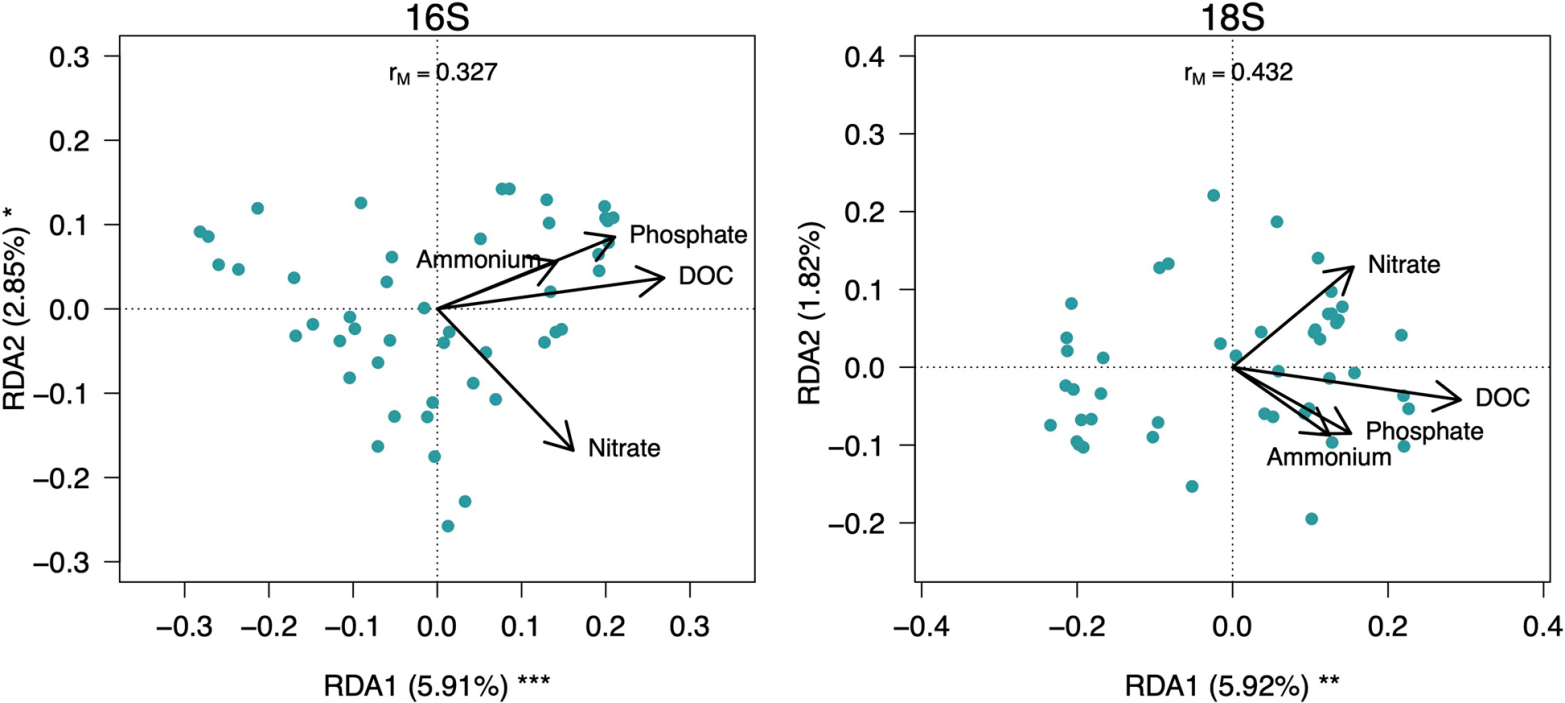
Biplots from RDA based on Hellinger-transformed ASVs (16S rRNA and 18S rRNA genes) and major nutrients content. The percentage of variance explained by each axis and its significance (***P < 0.001, **P < 0.01) are indicated. rM is the Mantel correlation coefficient between the Hellinger distance among samples and the Euclidean distance among the corresponding symbols in the graph

The contribution of the levels of nutrients (NH_4_^+^, NO_3_^−^, and PO_4_^3−^, and DOC) on the community structure of bacteria and eukaryotes inhabiting snow algae blooms was assessed through an RDA model and ANOVA-like permutation tests. Our results showed that the bacterial community structure was significantly affected by the levels of NO_3_^−^ (*F*_1,41_ = 2.1012, *P* = 0.011) and DOC (*F*_1,41_ = 1.9364, *P* = 0.016), while eukaryote community structure was affected significantly by the levels of DOC (*F*_1,41_ = 2.9688, *P* = 0.003). However, it must be mentioned that our RDA model captured a small percentage (bacteria = 7.9%; eukaryotes = 7.7%) of the variation of bacteria and eukaryotes (Fig. 6). The concentration of each nutrient in each snow sample is detailed in Supplementary Table 1.

### Ecological processes governing the microbial assembly in snow algae blooms

**Fig. 7:**
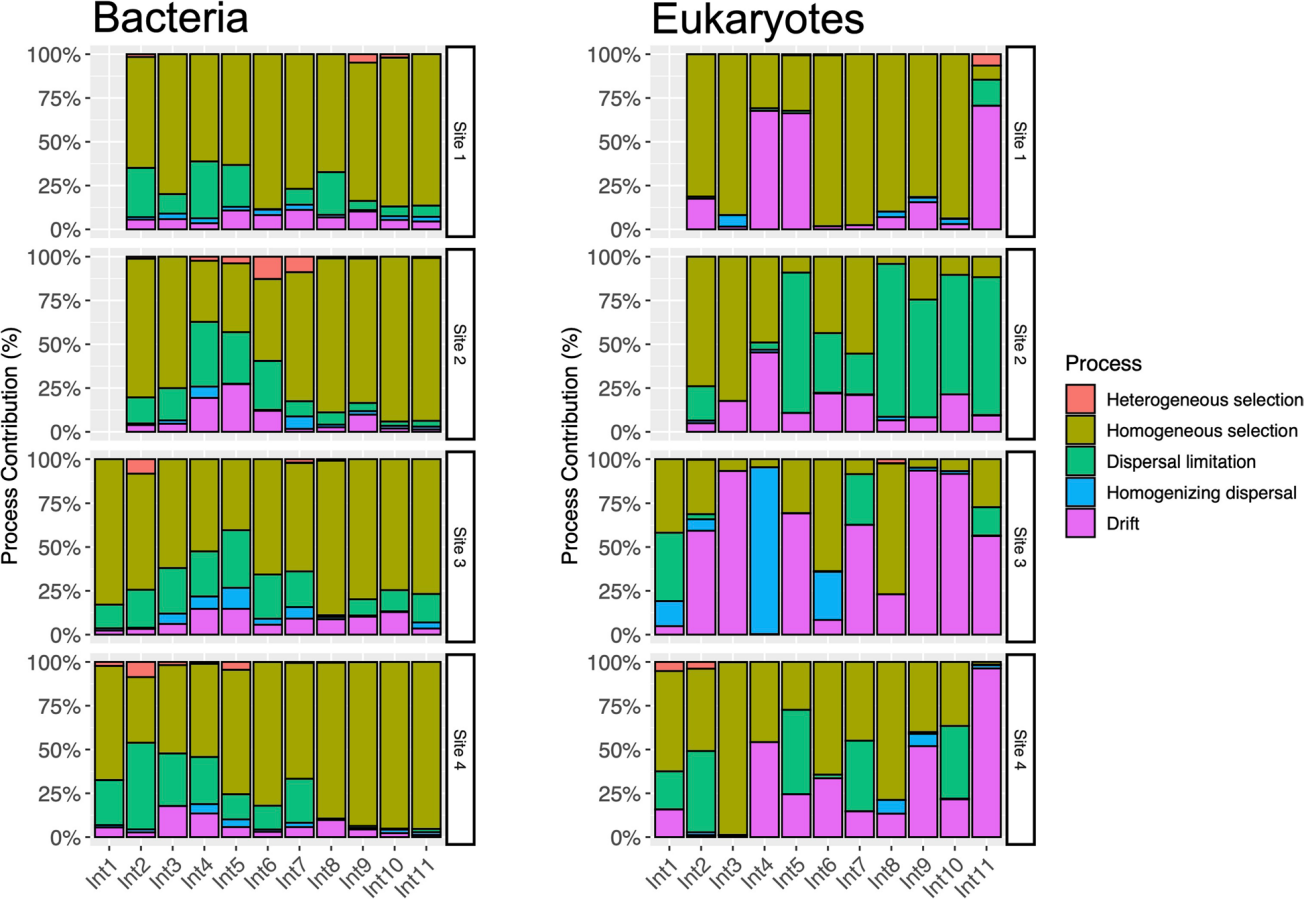
Relative importance of different ecological processes during the assembly of the bacterial and eukaryotic community. Bar plots indicate the process contribution when samples of two successive sampling dates were compared. Int*N*, *N* represents the interval of comparison. For instance, *N* = 1 means comparisons between sampling days 1 and 2

Based on the principle of the null models employed by iCAMP, our results show that both deterministic and stochastic processes drive the temporal variation of snow algae blooms. However, the contribution of each ecological process differed between the bacterial and the eukaryotic communities. For bacteria, the estimated importance of stochasticity (dispersal limitation, homogenizing dispersal, and drift) during the formation of algae blooms ranged between 12 and 48% (Table 2). Among these processes, dispersal limitation contributed the most to the assembly of the community, but its contribution declined from a mean value of 30%, recorded between December 4 and December 7 (Int 4), to 7% at the end of the field experiment (Int 11) (Table 2). This decline in the contribution of the stochastic process was observed in the four study sites (Fig. 7). In contrast, the importance of deterministic selection (homogeneous and heterogeneous selection) reached its peak between December 26 and December 29 (Int 11) accounting for 88% and its lowest level (50%) between December 4 and December 7 (Int 4) (Table 2). Finally, homogeneous selection was the selective process with the highest contribution in all the study sites (Fig. 7). In the case of the eukaryotic community, changes in diversity due to stochasticity varied between 30 and 87% (Table 3). Dispersal limitation and drift were the two processes that most contributed to stochasticity. In the case of dispersal limitation, its contribution was more noticeable on site 2 (Fig. 7), and in the case of drift, its contribution was more prominent on sites 3 and 4 (Fig. 7).

**Table 2:** Contribution of different ecological processes to the assembly of the bacterial communities within snow algae blooms. IntN represents the interval of comparison between consecutive sampling days

***Interval***	**Heterogeneous selection**		**Homogeneous selection**		**Dispersal limitation**		**Homogenizing dispersal**		**Drift**	
	*Mean*	*sd*	*Mean*	*sd*	*Mean*	*sd*	*Mean*	*sd*	*Mean*	*sd*
*Int1*	1%	± 2%	74%	± 13%	20%	± 9%	1%	± 0%	4%	± 2%
*Int2*	5%	± 4%	62%	± 17%	29%	± 15%	1%	± 0%	4%	± 1%
*Int3*	0%	± 1%	67%	± 13%	21%	± 8%	3%	± 2%	9%	± 6%
*Int4*	1%	± 1%	50%	± 11%	30%	± 5%	5%	± 2%	13%	± 7%
*Int5*	2%	± 2%	54%	± 16%	25%	± 8%	5%	± 5%	15%	± 9%
*Int6*	3%	± 6%	71%	± 19%	17%	± 13%	2%	± 1%	7%	± 4%
*Int7*	3%	± 4%	70%	± 7%	16%	± 8%	5%	± 2%	7%	± 4%
*Int8*	1%	± 0%	83%	± 10%	8%	± 11%	1%	± 0%	7%	± 3%
*Int9*	2%	± 2%	84%	± 7%	5%	± 3%	1%	± 1%	9%	± 3%
*Int10*	0%	± 10%	87%	± 10%	5%	± 5%	2%	± 1%	6%	± 5%
*Int11*	0%	± 0%	88%	± 8%	7%	± 6%	2%	± 1%	3%	± 2%

**Table 3:** Contribution of different ecological processes to the assembly of the eukaryote communities within snow algae blooms. IntN represents the interval of comparison between consecutive sampling days

***Interval***	**Heterogeneous selection**		**Homogeneous selection**		**Dispersal limitation**		**Homogenizing dispersal**		**Drift**	
	*Mean*	*sd*	*Mean*	*sd*	*Mean*	*sd*	*Mean*	*sd*	*Mean*	*sd*
*Int1*	3%	± 4%	50%	± 11%	30%	± 12%	7%	± 10%	10%	± 8%
*Int2*	1%	± 2%	58%	± 23%	17%	± 21%	3%	± 3%	21%	± 27%
*Int3*	0%	± 0%	70%	± 43%	0%	± 0%	2%	± 3%	28%	± 44%
*Int4*	0%	± 0%	33%	± 20%	1%	± 2%	24%	± 47%	42%	± 29%
*Int5*	0%	± 0%	25%	± 11%	32%	± 39%	0%	± 0%	43%	± 30%
*Int6*	0%	± 0%	67%	± 22%	9%	± 17%	7%	± 14%	16%	± 14%
*Int7*	0%	± 0%	52%	± 37%	23%	± 17%	0%	± 0%	25%	± 26%
*Int8*	1%	± 1%	62%	± 39%	22%	± 44%	3%	± 3%	12%	± 8%
*Int9*	0%	± 0%	38%	± 33%	17%	± 33%	3%	± 3%	42%	± 39%
*Int10*	0%	± 0%	37%	± 40%	28%	± 33%	1%	± 1%	34%	± 39%
*Int11*	2%	± 3%	12%	± 11%	28%	± 35%	1%	± 1%	58%	± 37%

In addition, the importance of the ecological processes governing the assembly of the eukaryotic communities fluctuates during the formation of the algae blooms. For instance, the contribution of homogeneous selection declined as the formation of the blooms progressed, reaching its highest level between December 2 and December 4 (Int 3) and its lowest level (12%) between December 26 and December 29 (Int 11) (Table 3). Likewise, the importance of drift increased from 10%, at the beginning of the field experiment, to ~58% towards the end of December (Int 11). However, this increase was not linear, and the importance of drift fluctuated over time in every study site (Fig. 7).

### Taxonomy of phylogenetic bins groups and potential biotic interactions

In this study, bacterial and eukaryotic taxa were classified into 45 and 19 phylogenetic bins, respectively. The top ten most abundant bins are detailed in Table 4.

**Table 4:** Top 10 most abundant phylogenetic bins. Taxonomy indicates the most representative taxa within each bin at its most resolved level according to Silva (bacteria) and PR2 (eukaryotes) databases. The complete list of taxa for all bins can be found in Supplementary Tables 3 and 4

**Bacteria**			**Eukaryotes**		
Bin ID	Relative abundance (%)	Taxonomy	Bin ID	Relative abundance (%)	Taxonomy
Bin17	12.9	*Polaromonas*	Bin4	37.7	*Chloromonas*
Bin1	9.9	*Psychrobacter*	Bin13	16.7	Chytridiomycetes
Bin37	9.2	*Flavobacterium*	Bin5	12.2	Chlamydomonadales_X
Bin10	7.3	*Marinobacter*	Bin8	11.0	*Chlamydomonas*
Bin15	5.1	*Rhodoferax*	Bin7	8.1	Prasiolales
Bin7	4.4	*Psychrobacter*	Bin9	2.7	Dothideomycetes
Bin38	4.4	*Flavobacterium*	Bin12	2.4	Pucciniomycotina
Bin34	4.4	Chloroplast	Bin17	2.1	Sandonidae
Bin30	3.6	*Cryobacterium*	Bin16	1.9	Sandonidae
Bin35	2.6	Chloroplast	Bin6	1.2	Ulotrichales

**Fig. 8:**
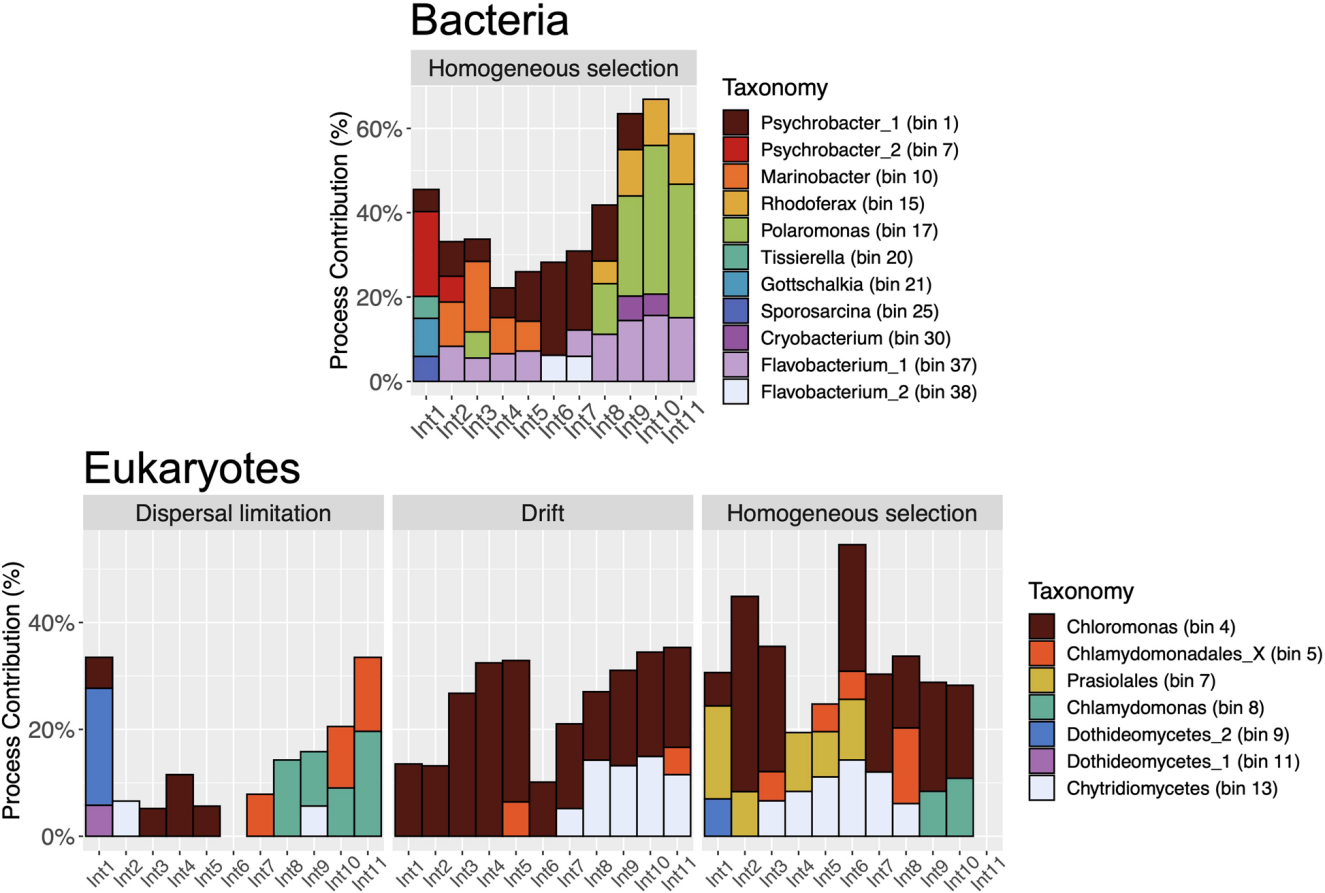
Contribution of bacterial and eukaryotic taxa to the ecological processes governing the community assembly. Bar plots represent the contribution of different ecological processes when samples of successive sampling dates were compared and designated as Int*N*. *N* represents the interval of comparison. For instance, *N* = 1 means a comparison between sampling days 1 and 2. For illustrative purposes, only taxa whose contribution was > 5% were considered. Bin’s taxonomy is detailed in Supplementary Tables 3 and 4

Our results showed that bacteria such as *Flavobacterium* (bins 37 and 38), *Polaromonas* (bin 17), *Marinobacter* (bin 10), and *Psychrobacter* (bins 1 and 7) had the highest contribution to homogeneous selection. *Flavobacterium* bacteria impacted the microbial assembly during the whole period of snow algae blooms formation, increasing their contribution towards the end of the field experiment (Fig. 8). In addition, the influence of *Psychrobacter* and *Marinobacter* to homogenous selection was greater at the early stages of the bloom formation, contrasting with *Polaromonas* bacteria that played a more prominent role in the microbial assembly during the last half of December.

The assembly of eukaryotic communities of snow algae blooms was mostly defined by dispersal limitation, drift, and homogenous selection. The greatest contributions to dispersal limitation were from algal species classified into the genus *Chlamydomonas* (bin 8) and into the order Chlamydomonadales_X (bin 5). In contrast, algae of the genus *Chloromonas* (bin 4) and the order Prasiolales (bin 7) could be mostly associated with drift and homogenous selection (Fig. 8). Finally, fungi of the family Chytridiomycetes (bin 13) impacted drift and homogenous selection. The most representative taxon within the phylogenetic bin of *Chloromonas* is part of the group classified as *Chloromonas* D, while the most representative taxon of *Chlamydomonas* was part of the group named uncultured Chlamydomonadales (Supplementary Tables 2 and 4). In the case of the bin denominated Chlamydomonadales_X, the most abundant taxon was part of *Chlorominina*. Finally, within the bin of Prasiolales, the most representative taxon could not be classified deeper than order level and belongs to Prasiolales A (Supplementary Tables 2 and 4).

Correlations between phylogenetic bins that contributed to the microbial assembly revealed significant correlations between several eukaryotic and bacterial taxa. For instance, Dothideomycetes fungi were positively correlated with Prasiolales algae and bacteria of the genera *Tissierella*, *Gottschalkia*, *Sporosarcina*, and *Psychrobacter*. In contrast, Chytridiomycetes were negatively correlated with the same algal and bacterial taxa (Supplementary Fig. 3).

Regarding algae taxa, the bin of *Chloromonas* negatively correlated with other algal bins and with bacteria of the genus *Rhodoferax*. The only positive correlation of this bin was with the bacterial genus *Marinobacter*. Likewise, bins Chamydomonadales_X and *Chlamydomonas* did not correlate with other algal taxa, and in the case of Chamydomonadales_X, it did not correlate with any other taxa, while *Chlamydomonas* positively correlate with bacteria of the genera *Rhodoferax* and *Polaromonas*.

In addition, bacteria-bacteria correlated taxa were characterized by positive correlations between bacteria of the genera *Polaromonas*, *Rhodoferax*, *Cryobacterium*, and *Flavobacterium* which dominated the colored blooms. Likewise, positive correlations between bacteria of the genera *Tissierella*, *Gottschalkia*, *Sporosarcina*, and *Psychrobacter*, which were more abundant in the early stages of algae blooms, were detected.

## Discussion

During the 2019/2020 Austral summer, exceptionally high temperatures were recorded across Antarctica, leading to the melting of ice and exposure of new ice-free areas [58]. These exceptional conditions were driven by anomalously long periods of sustained air temperatures ≥ 0 °C (55 to 90 h) from late November onwards, which affected meltwater refreezing [2]. Our results showed that at the Isabel Riquelme islet (in the northern part of the West Antarctic Peninsula), these warm conditions led to a fast development of snow algae blooms during December 2019. Moreover, along with the establishment of snow algae blooms, we also observed a rapid color change from green to red in the lapse of a two-week period. Thus, as previously reported, the appearance of snow algae was likely determined by periods of not abundant snowfalls and air temperatures above the melting point [48].

In this rapid progression of the snow algae bloom formation, we could record the temporal variation in the microbial composition and the dynamics of the main mechanisms that governed the community assembly highlighting that both the taxonomic groups and the ecological process change with time.

### Dominant snow algae within blooms vary even between nearby sites

Microbial communities within Antarctic snow algae blooms have been described as heterogeneous and highly variable even in closely related snowpacks are compared [64]. In this study, we provide a temporal perspective on the composition of snow algae blooms highlighting the differences in the process that led to the taxonomic configuration of their microbial community.

The molecular survey performed in the four study sites at Isabel Riquelme Islet revealed the presence of four different blooms characterized by distinct species of snow algae. Site 1 was a bloom that started green but turned into red and was dominated by *Chloromonas* classified into group D (Supplementary Table 2). *Chloromonas* D is a group formed by sequences obtained from algae isolated previously by Segawa et al. [59] in red snow samples from Antarctica (Otu025, [59]) and Alaska (Otu005, [59]). The second site that formed a red bloom (site 3) was dominated by algae classified into the group *Chloromonas* B, with a sparse presence of algae classified into groups *Chloromonas* A, and *Chloromonas* D. Taxonomically, the group *Chloromonas* B is formed by species isolated from red snow, orange snow from localities that included Antarctica, Czech Republic, Poland, and Japan. In the case of *Chloromonas* A, it gathered almost the same sequences that *Chloromonas* B, but also included several sequences of the study of Matsuzaki et al. [41]. The multiple geographical sources of these algae highlight their cosmopolitan distribution that includes polar and sub-polar environments. This contrasts with the *Chloromonas* D group, which has been only described in polar environments.

On sites 2 and 4, we observed only green algae blooms. In the early stages of the bloom on site 2, algae of the groups *Chloromonas* A, B, and C were detected; however, the advanced stage of the blooms was dominated by algae of the group uncultured Chlamydomonadales. Regarding the latter algae group, the best hit after aligning the sequences against the Nucleotide collection (nr/nt) using BLAST, returned the sequence of a microorganism isolated from lake water in Luxemburg (Accession: GU067821.1). With no more information available, we propose that this finding emphasizes the incomplete knowledge regarding the true diversity of snow algae blooms, especially in sites with limited access, like Antarctica. Likewise, the bloom on site 4 was dominated by algae of the groups *Chloromonas* B and D in the early stages, but as the bloom progressed, algae classified into the groups *Chlorominima*, *Chlamydomonas* A, *Microglena*, and uncultured Chlamydomonadales increased their abundances. Therefore, contrasting with the blooms in the other study sites, this bloom was a mix of algal species with not a clear consistent dominance of none of them. The group *Chlorominima* gathered sequences isolated mostly from Antarctica, except for one of them that was obtained from freshwater from the Arctic (Accession: KU886306.1). The Antarctic sources include red snow (LC371433.1, [59]; MW553075.1, [18]), freshwater (JQ926737.1), and snow without additional information about the color (HQ404890.1). In the case of the group *Chlamydomonas* A, the best BLAST hit returned a single sequence (Accession: LC371424.1, [59]) obtained from a red snow sample from Antarctica. Based on these findings, the bloom on site 4 was dominated by algae species likely endemic to Antarctica. Finally, algae within the group *Microglena* appeared in the snow samples collected on December 26 and December 29. This group was formed by algal taxa isolated from diverse Antarctic and Arctic ecosystems such as sea ice, snow, and freshwater.

### Deterministic processes govern the assembly of bacterial communities

During the formation of snow algae blooms, the bacterial community assembly was mostly governed by selective pressure resulting from consistent environmental conditions (homogeneous selection). One of the environmental factors that might impose a selective pressure on the community is the availability and levels of nutrients. For instance, phosphorus has commonly been regarded as a major nutrient controlling freshwater productivity, whereas nitrogen drives the productivity of marine coastal waters [12, 49]. However, in this study, we showed that although DOC and NO_3_^−^ impacted the community structure of bacteria inhabiting snow algae blooms, their contribution was low. During the summer season, some coastal snowfields across the Maritime Antarctic receive a high input of these nutrients due to the presence of penguin colonies [1]. In fact, due to their role as nutrient reservoirs and sites with highly dynamic carbon flux, coastal snowpacks have been regarded as “power plants” of microbial diversity [46]. In our study, values of, NH_4_^+^, NO_3_^−^, and PO_4_^3−^ for green blooms averaged 3.7 ± 3.9 (mean ± sd), 1 ± 1, and 4.3 ± 3.6 mg/L, respectively. While for red blooms, the mean values are close to 5.4 ± 8.5, 0.5 ± 0.5, and 6.2 ± 6.6 mg/L, respectively. These values are 10 times higher than those recorded in other snowpacks from Antarctica [46]. Therefore, it could consistently be argued that snow algae blooms in this region flourish in a macronutrient-rich environment and the variation of nutrient levels has a minor role in the assembly of the bacterial community.

Another possible selective force is environmental stressor filtering. This type of filtering selects related taxa that share evolutionarily conserved traits that allow them to withstand harsh environmental factors, while taxa lacking these traits fail to become established [25]. It has been reported that bacteria inhabiting snow algae blooms share a consistent functional profile with features related to cold environments [64], thus factors such as low temperatures, high solar radiation, changing water availability, play a major role in selecting the species that configure the bacterial community [64].

Selective pressures also arise from biotic interactions [3] and highly abundant microbial taxa may inhibit other microbes or may benefit from positive interactions with different community members [11]. Our results showed that bacteria diversity and richness did not vary significantly during the formation of the blooms. However, as snow algae increased their abundance, the bacterial community was dominated by a couple of taxa frequently reported in snow algae blooms such as *Polaromonas* and *Flavobacterium* [13, 65, 72]. Here, bacterial taxa that significantly contributed to the assembly of the community such as *Polaromas* correlated positively with *Chlamydomonas*, as well as bacteria of the genera *Cryobacterium*, suggesting potential biotic interactions further to be explored.

### Stochastic processes govern the assembly of eukaryotic communities

Despite the contribution of selective forces (homogenous selection) to the assembly of the eukaryotic community in snow algae blooms, stochasticity (dispersal limitation and drift) can be regarded as the most relevant factor. Dispersal is predominantly passive [44] and when selection pressure is weak, and the dispersal rate between communities is limiting, the turnover dynamics within a community are subject to stochastic changes in species abundance and random variations in population [16].

At first, this finding might be contradictory because the ubiquitous presence of snow algal species across different cryospheric ecosystems supports the idea that there is a global dispersal mechanism influencing the distribution of these organisms [7, 59]. Moreover, snow algae have a consistent functional profile related with the tolerance to the environmental stressors in cold habitats [18, 53, 54, 64] and it has been proposed that highly specialized taxa may be more likely to be cosmopolitan [6, 14]. However, regarding their proliferation patterns, it has been shown that snow algae colonize discrete snow patches producing colorful blooms and that each species within a patch is highly clonal [8].

A key factor reconciling the cosmopolitan nature of snow algae and the dispersal limitation recorded in this study might be their life cycle. Snow algae of the order Chlamydomonadales have a distinct life cycle consisting of a vegetative flagellate stage and an immobile cyst or spore stage [56]. The encysted phase of snow algae is characterized by the accumulation of secondary carotenoids that may act as photoprotective compounds by absorbing visible wavelengths and dissipating the excess radiant energy as heat [4, 15]. In addition, encysted cells have a high abundance of storage and reserve metabolites likely to face stressful conditions [37]. In contrast, during the vegetative phase of snow algae, their metabolic profile is characterized by key metabolites involved in growth and proliferation and the dominant pigment is chlorophyll.

During the melting season, snow algae usually germinate from underlying resting spores on the ground surface that have been dormant from the previous melting season [27, 29]. The formation of dormant cysts is likely a key factor in the dispersion of snow algae [39]. In fact, dispersion has been regarded as a pivotal mechanism connecting very distant communities and is particularly relevant for microorganisms with dormant phases in their life cycle [43]. Moreover, studies carried out on the *Raphidonema* complex appear to indicate that ancestral cosmopolitan phylotypes dispersed globally and that following microevolutive processes, the actual endemic phylotypes found in different places were formed [60]. Thus, our findings confirm a diversity marked by endemic groups, which could show dispersal limitation and be governed by drift when their cells are in a vegetative state. In contrast, the presence of dormant phases in some groups could be related with cosmopolitan features.

Finally, it has been pointed out that the increasing temperatures in the West Antarctic Peninsula along with the nutrient availability are key factors determining the distribution of green snow algae in this region [20]. As the 0 °C isotherm is predicted to increase its elevation, temperatures above 0 °C would occur more frequently in the southern areas of the Antarctic Peninsula [62]. This condition in addition with higher temperatures that increase the number of airborne bacteria and fungi [24, 61], and eventually increase the airborne snow algae, might open spaces for the colonization of new areas. However, the dispersal of snow algae might be limited to certain life stages able to cope with the environmental factors that influence airborne microbiota such as wind speed, temperature, and exposure to UV light and solar radiation [50].

### Integrative model explaining the microbial assembly in snow algae blooms

**Fig. 9:**
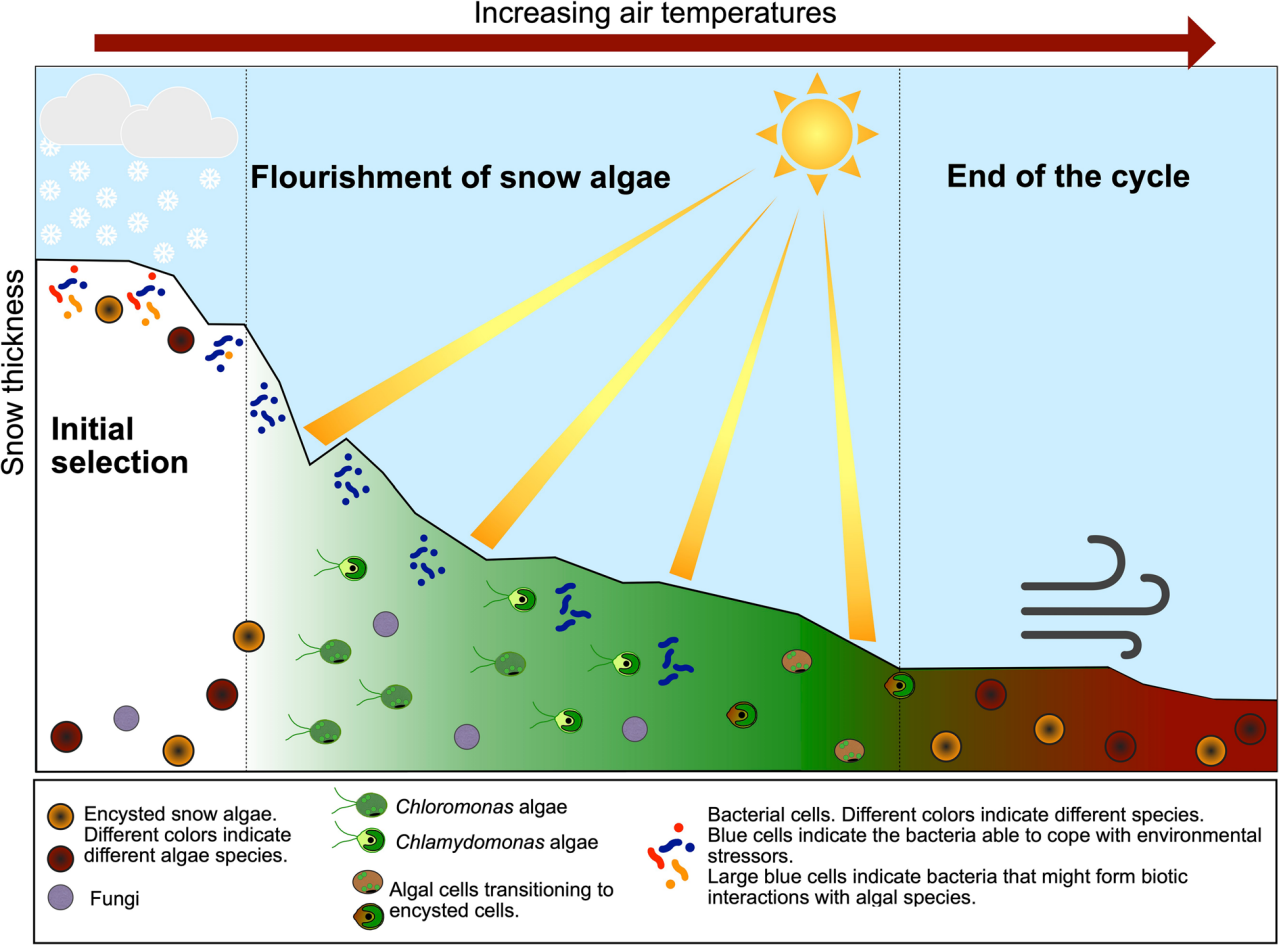
Proposed model for the assembly of Antarctic snow algae communities

Our results revealed that the assembly of bacterial and eukaryotic communities within snow algae blooms is driven by different ecological mechanisms. At first, we propose that snowing events allow the eolian deposition of microorganisms. Then, in the case of bacteria, the first selective force is likely the environmental filtering that selects taxa highly adapted regardless that they are less likely to have dispersal barriers. Thus, post-depositional selection occurs because factors like low temperatures, rapid osmotic shifts, and high radiation filter those taxa able to tolerate these abiotic stressors. In addition, snow algae cells remain dormant until the environmental conditions become favorable for their flourishing. In a macronutrient-rich environment, selection is driven by the constant environment constraints present in Antarctic snow (initial selection, Fig. 9). In the context of climate change, the homogenous environmental conditions that select microorganisms might change (e.g., warmer temperatures), affecting the composition of the bacterial community. Thus, bacterial species that would otherwise be selected out from the community might now proliferate in less challenging environmental conditions. In the second stage, snow melting increases the water availability that along with high radiation during the spring-summer season and allows the development of snow algae. Consequently, encysted dormant cells change to vegetative and increase their abundance forming colored blooms. Although snow algae should cope with harsh environmental conditions, selective forces related with stress filtering are weaker at this stage than processes such as dispersion limitation and drift. When algal propagules successfully establish under favorable conditions, they reproduce asexually with a low dispersion potential in this vegetative stage. Alternatively, snow algae flourishing may be a result of the reestablishment of algae in situ from the previous growing season (flourishment of snow algae, Fig. 9). In contrast, bacteria remain governed by selective pressure with the potential of generating biotic interactions (e.g., *Chlamydomonas* algae and *Polaromonas* bacteria). Meanwhile, fungal species increase their abundance probably because of the high nutrient availability.

Finally, after snow algae complete their life cycle, cells increase the production and accumulation of secondary carotenoids, lose their flagella, and become dormant. In this state, snow algae may remain in the same site until the next melting season ([52, 57]) or be air transported to other sites (end of the cycle, Fig. 9). Therefore, despite the increasing occurrence of snow algae blooms in maritime Antarctica, these microbial communities are threatened if the snow melts faster than the transition of snow algae from vegetative cells to dormant propagules.

### Final remarks

The study of snow algae has shown notable advances during the last couple of years. However, ecological aspects regarding their community assembly and dispersion of snow algae remain in an early stage of development compared with the knowledge about the physiology, distribution, and diversity of snow algae species.

In this study, using amplicon sequencing, we addressed the ecological question of how the community of bacteria and eukaryotes progresses over time from early stages to colorful algae blooms. Despite that the number of independent experiments is limited and were performed in only one site, our findings and the proposed model can be used to generate further hypotheses regarding the environmental constraints influencing the establishment of snow algae communities. In addition, it opens questions about the role and relevance of the physiological state of snow algae (red/encysted cells versus green/vegetative cells) in the dispersal and occurrence of algae blooms.

Finally, we acknowledge that the findings of this study might be constrained to the specific environmental features of Antarctic snow ecosystems such as high nutrient content or the presence of endemic snow algae species. Therefore, the replication of a temporal series study in different sites and conditions is a necessary next step for the understanding of the ecological processes governing the assembly of snow algae communities globally.

### Supplementary Information

**Additional file 1: Supplementary Fig. 1.** Rarefaction curves of bacterial and eukaryotic (16S rRNA and 18S rRNA genes) ASVs. **Supplementary Fig. 2.** Shannon-Wiener diversity index and Richness (Chao 1) of bacterial and eukaryotic communities in snow samples collected at O’Higgins. **Supplementary Fig. 3.** Matrix for the correlations between the abundances of bacteria and eukaryotes. The figure illustrates positive (blue) and negative (red) significant correlations between taxa that most contributed to the assembly of the microbial community. The size of circles is proportional to the correlation value. Only significant correlations (*p* < 0.05) are illustrated.

**Additional file 2: Supplementary Table 1.** Overview of snow samples description and coordinates and nutrients content. The method for DOC quantification records DOC concentrations <=20 mg/L.

**Additional file 3: Supplementary Table 2.** BLAST results for the ASVs of the classes Trebouxiophyceae, and Chlorophyceae. ASVs with identical hits were grouped together and named after the most distinctive/common taxon. When there was none clear taxonomic pattern, groups were named after the most resolved taxonomic level according to the PR2 database.

**Additional file 4: Supplementary Table 3.** Composition of phylogenetic-bins used to infer bacterial community assembly mechanisms. The table shows every ASV Silva taxonomy and the best BLAST hit of the three most abundant ASVs against the Nucleotide collection (nr/nt) database.

**Additional file 5: Supplementary Table 4.** Composition of phylogenetic-bins used to infer eukaryotes community assembly mechanisms. The table shows every ASV PR2 taxonomy and the best BLAST hit of the three most abundant ASVs against the Nucleotide collection (nr/nt) database.

## Data Availability

Raw metabarcoding snow datasets are deposited in the European Nucleotide Archive under study number PRJEB55994.

## References

[1] Abakumov E (2018). Content of available forms of nitrogen, potassium and phosphorus in ornithogenic and other soils of the Fildes Peninsula (King George Island, Western Antarctica). Biol Commun.

[2] Banwell AF, Datta RT, Dell RL, Moussavi M, Brucker L, Picard G, Shuman CA, Stevens LA (2021). The 32-year record-high surface melt in 2019/2020 on the northern George VI Ice Shelf, Antarctic Peninsula. Cryosphere.

[3] Benkman CW (2013). Biotic interaction strength and the intensity of selection. Ecol Lett.

[4] Bidigare RR, Ondrusek ME, Kennicutt MC, Iturriaga R, Harvey HR, Hoham RW, Macko SA (1993). Evidence a photoprotective for secondary carotenoids of snow algae^1^. J Phycol.

[5] Bodenhofer U, Bonatesta E, Horejš-Kainrath C, Hochreiter S (2015). msa: an R package for multiple sequence alignment. Bioinformatics.

[6] Brown JK, Hovmøller MS (2002). Aerial dispersal of pathogens on the global and continental scales and its impact on plant disease. Science.

[7] Brown SP, Jumpponen A (2019). Microbial ecology of snow reveals taxa-specific biogeographical structure. Microb Ecol.

[8] Brown SP, Ungerer MC, Jumpponen A (2016). A community of clones: snow algae are diverse communities of spatially structured clones. Int J Plant Sci.

[9] Callahan BJ, McMurdie PJ, Rosen MJ, Han AW, Johnson AJA, Holmes SP (2016). DADA2: high-resolution sample inference from Illumina amplicon data. Nat Methods.

[10] Cheung MK, Au CH, Chu KH, Kwan HS, Wong CK (2010). Composition and genetic diversity of picoeukaryotes in subtropical coastal waters as revealed by 454 pyrosequencing. ISME J.

[11] Cosetta CM, Wolfe BE (2019). Causes and consequences of biotic interactions within microbiomes. Curr Opin Microbiol.

[12] Crain CM (2007). Shifting nutrient limitation and eutrophication effects in marsh vegetation across estuarine salinity gradients. Estuaries Coasts.

[13] Darcy JL, Lynch RC, King AJ, Robeson MS, Schmidt SK (2011). Global distribution of Polaromonas phylotypes-evidence for a highly successful dispersal capacity. PLoS One.

[14] Davison J, Moora M, Öpik M, Adholeya A, Ainsaar L, Bâ A, Burla S, Diedhiou AG, Hiiesalu I, Jairus T (2015). Global assessment of arbuscular mycorrhizal fungus diversity reveals very low endemism. Science.

[15] Dial RJ, Ganey GQ, Skiles SM (2018). What color should glacier algae be? An ecological role for red carbon in the cryosphere. FEMS Microbiol Ecol.

[16] Evans S, Martiny JB, Allison SD (2017). Effects of dispersal and selection on stochastic assembly in microbial communities. ISME J.

[17] Fadrosh DW, Ma B, Gajer P, Sengamalay N, Ott S, Brotman RM, Ravel J (2014). An improved dual-indexing approach for multiplexed 16S rRNA gene sequencing on the Illumina MiSeq platform. Microbiome.

[18] Gálvez FE, Saldarriaga-Córdoba M, Huovinen P, Silva AX, Gómez I (2021). Revealing the characteristics of the Antarctic snow alga Chlorominima collina gen. et sp. nov. through taxonomy, physiology, and transcriptomics. Front Plant Sci.

[19] Ganey GQ, Loso MG, Burgess AB, Dial RJ (2017). The role of microbes in snowmelt and radiative forcing on an Alaskan icefield. Nat Geosci.

[20] Gray A, Krolikowski M, Fretwell P, Convey P, Peck LS, Mendelova M, Smith AG, Davey MP (2020). Remote sensing reveals Antarctic green snow algae as important terrestrial carbon sink. Nat Commun.

[21] Gu Z, Liu K, Pedersen MW, Wang F, Chen Y, Zeng C, Liu Y (2021). Community assembly processes underlying the temporal dynamics of glacial stream and lake bacterial communities. Sci Total Environ.

[22] Guillou L, Bachar D, Audic S, Bass D, Berney C, Bittner L, Boutte C, Burgaud G, de Vargas C, Decelle J, del Campo J, Dolan JR, Dunthorn M, Edvardsen B, Holzmann M, Kooistra WHCF, Lara E, Le Bescot N, Logares R, Mahé F, Massana R, Montresor M, Morard R, Not F, Pawlowski J, Probert I, Sauvadet A-L, Siano R, Stoeck T, Vaulot D, Zimmermann P, Christen R (2013). The Protist Ribosomal Reference database (PR2): a catalog of unicellular eukaryote small sub-unit rRNA sequences with curated taxonomy. Nucleic Acids Res.

[23] Harrell FE, Harrell Jr MFE (2019). Package ‘hmisc’. CRAN2018.

[24] Harrison RM, Jones AM, Biggins PD, Pomeroy N, Cox CS, Kidd SP (2005). Climate factors influencing bacterial count in background air samples. Int J Biometeorol.

[25] HilleRisLambers J, Adler PB, Harpole WS, Levine JM, Mayfield MM (2012). Rethinking community assembly through the lens of coexistence theory. Annu Rev Ecol Evol Syst.

[26] Hodson AJ, Nowak A, Cook J, Sabacka M, Wharfe ES, Pearce DA, Convey P, Vieira G (2017). Microbes influence the biogeochemical and optical properties of maritime Antarctic snow. J Geophys Res Biogeosci.

[27] Hoham RW, Berman JD, Rogers HS, Felio JH, Ryba JB, Miller PR (2006). Two new species of green snow algae from Upstate New York, *Chloromonas chenangoensis* sp. nov. and *Chloromonas tughillensis* sp. nov. (Volvocales, Chlorophyceae) and the effects of light on their life cycle development. Phycologia.

[28] Huovinen P, Ramírez J, Gómez I (2018). Remote sensing of albedo-reducing snow algae and impurities in the Maritime Antarctica. ISPRS J Photogramm Remote Sens.

[29] Jones HG, Pomeroy JW, Walker DA, Hoham RW. Snow ecology: an interdisciplinary examination of snow-covered ecosystems. Cambridge: Cambridge University Press; 2001.

[30] Kazamia E, Czesnick H, Nguyen TTV, Croft MT, Sherwood E, Sasso S, Hodson SJ, Warren MJ, Smith AG (2012). Mutualistic interactions between vitamin B12-dependent algae and heterotrophic bacteria exhibit regulation. Environ Microbiol.

[31] Kindt R, Kindt MR. Package ‘BiodiversityR.’ R Proj. 2015.

[32] King JC (1994). Recent climate variability in the vicinity of the Antarctic Peninsula. Int J Climatol.

[33] Klindworth A, Pruesse E, Schweer T, Peplies J, Quast C, Horn M, Glöckner FO (2013). Evaluation of general 16S ribosomal RNA gene PCR primers for classical and next-generation sequencing-based diversity studies. Nucleic Acids Res.

[34] Krug L, Erlacher A, Markut K, Berg G, Cernava T (2020). The microbiome of alpine snow algae shows a specific inter-kingdom connectivity and algae-bacteria interactions with supportive capacities. ISME J.

[35] Legendre P, Gallagher ED (2001). Ecologically meaningful transformations for ordination of species data. Oecologia.

[36] Lutz S, Anesio AM, Edwards A, Benning LG (2017). Linking microbial diversity and functionality of arctic glacial surface habitats. Environ Microbiol.

[37] Lutz S, Anesio AM, Field K, Benning LG (2015). Integrated ‘omics’, targeted metabolite and single-cell analyses of Arctic snow algae functionality and adaptability. Front Microbiol.

[38] Makoto K, Wilson SD (2019). When and where does dispersal limitation matter in primary succession?. J Ecol.

[39] Marshall WA, Chalmers MO (1997). Airborne dispersal of Antarctic terrestrial algae and cyanobacteria. Ecography.

[40] Marshall GJ, Lagun V, Lachlan-Cope TA (2002). Changes in Antarctic Peninsula tropospheric temperatures from 1956 to 1999: a synthesis of observations and reanalysis data. Int J Climatol.

[41] Matsuzaki R, Kawai-Toyooka H, Hara Y, Nozaki H (2015). Revisiting the taxonomic significance of aplanozygote morphologies of two cosmopolitan snow species of the genus Chloromonas (Volvocales, Chlorophyceae). Phycologia.

[42] McMurdie PJ, Holmes S (2013). phyloseq: an R package for reproducible interactive analysis and graphics of microbiome census data. PLoS One.

[43] Mestre M, Höfer J (2021). The microbial conveyor belt: connecting the globe through dispersion and dormancy. Trends Microbiol.

[44] Nemergut DR, Schmidt SK, Fukami T, O’Neill SP, Bilinski TM, Stanish LF, Knelman JE, Darcy JL, Lynch RC, Wickey P (2013). Patterns and processes of microbial community assembly. Microbiol Mol Biol Rev.

[45] Ning D, Yuan M, Wu L, Zhang Y, Guo X, Zhou X, Yang Y, Arkin AP, Firestone MK, Zhou J (2020). A quantitative framework reveals ecological drivers of grassland microbial community assembly in response to warming. Nat Commun.

[46] Nowak A, Hodson A, Turchyn A (2018). Spatial and temporal dynamics of dissolved organic carbon, chlorophyll, nutrients, and trace metals in Maritime Antarctic snow and snowmelt. Front Earth Sci.

[47] Oksanen J, Kindt R, Legendre P, O’Hara B, Stevens MHH, Oksanen MJ, Suggests M (2007). The vegan package. Commun Ecol Package.

[48] Onuma Y, Takeuchi N, Takeuchi Y (2016). Temporal changes in snow algal abundance on surface snow in Tohkamachi, Japan. Bull Glaciol Res.

[49] Paerl HW (2009). Controlling eutrophication along the freshwater–marine continuum: dual nutrient (N and P) reductions are essential. Estuaries Coasts.

[50] Pearce DA, Bridge PD, Hughes KA, Sattler B, Psenner R, Russell NJ (2009). Microorganisms in the atmosphere over Antarctica. FEMS Microbiol Ecol.

[51] Price MN, Dehal PS, Arkin AP (2010). FastTree 2–approximately maximum-likelihood trees for large alignments. PLoS One.

[52] Procházková L, Leya T, Krížková H, Nedbalová L. Sanguina nivaloides and Sanguina aurantia gen. et spp. nov. (Chlorophyta): the taxonomy phylogeny biogeography and ecology of two newly recognised algae causing red and orange snow. FEMS Microbiol Ecol. 2019;95(6):fiz064. 10.1093/femsec/fiz064. 10.1093/femsec/fiz064 PMC6545352 31074825

[53] Procházková L, Remias D, Bilger W, Křížková H, Řezanka T, Nedbalová L (2020). Cysts of the snow alga *Chloromonas krienitzii* (Chlorophyceae) show increased tolerance to ultraviolet radiation and elevated visible light. Front Plant Sci.

[54] Procházková L, Remias D, Holzinger A, Řezanka T, Nedbalová L (2018). Ecophysiological and morphological comparison of two populations of *Chlainomonas* sp. (Chlorophyta) causing red snow on ice-covered lakes in the High Tatras and Austrian Alps. Eur J Phycol.

[55] Quast C, Pruesse E, Yilmaz P, Gerken J, Schweer T, Yarza P, Peplies J, Glöckner FO (2012). The SILVA ribosomal RNA gene database project: improved data processing and web-based tools. Nucleic Acids Res.

[56] Remias D, Lütz C (2012). Cell structure and physiology of Alpine snow and ice algae. Plants in Alpine regions: cell physiology of adaption and survival strategies.

[57] Remias D, Procházková L, Holzinger A, Nedbalová L (2018). Ecology, cytology and phylogeny of the snow alga Scotiella cryophila K-1 (Chlamydomonadales, Chlorophyta) from the Austrian Alps. Phycologia.

[58] Robinson SA, Klekociuk AR, King DH, Pizarro Rojas M, Zúñiga GE, Bergstrom DM (2020). The 2019/2020 summer of Antarctic heatwaves. Glob Change Biol.

[59] Segawa T, Matsuzaki R, Takeuchi N, Akiyoshi A, Navarro F, Sugiyama S, Yonezawa T, Mori H (2018). Bipolar dispersal of red-snow algae. Nat Commun.

[60] Segawa T, Yonezawa T, Matsuzaki R, Mori H, Akiyoshi A, Navarro F, et al. Evolution of snow algae, from cosmopolitans to endemics, revealed by DNA analysis of ancient ice. ISME J. 2023;17(4):491–501. 10.1038/s41396-023-01359-3 PMC10030584 36650274

[61] Seino K, Takano T, Nakamura K, Watanabe M (2005). An evidential example of airborne bacteria in a crowded, underground public concourse in Tokyo. Atmos Environ.

[62] Siegert M, Atkinson A, Banwell A, Brandon M, Convey P, Davies B, et al. The Antarctic Peninsula under a 1.5 C global warming scenario. Front Environ Sci. 2019;7:102.

[63] SFS. Determination of chlorophyll *a* in water. Extraction with ethanol. Spectrophotometric method. SFS Standard 5772. 1993. p. 3. (in Finnish).

[64] Soto DF, Franzetti A, Gómez I, Huovinen P (2022). Functional filtering and random processes affect the assembly of microbial communities of snow algae blooms at Maritime Antarctic. Sci Total Environ.

[65] Soto DF, Fuentes R, Huovinen P, Gómez I (2020). Microbial composition and photosynthesis in Antarctic snow algae communities: integrating metabarcoding and pulse amplitude modulation fluorometry. Algal Res.

[66] Stegen JC, Lin X, Fredrickson JK, Konopka AE (2015). Estimating and mapping ecological processes influencing microbial community assembly. Front Microbiol.

[67] Sugimura Y, Suzuki Y (1988). A high-temperature catalytic oxidation method for the determination of non-volatile dissolved organic carbon in seawater by direct injection of a liquid sample. Mar Chem.

[68] Terashima M, Umezawa K, Mori S, Kojima H, Fukui M (2017). Microbial community analysis of colored snow from an Alpine snowfield in Northern Japan reveals the prevalence of Betaproteobacteria with snow algae. Front Microbiol.

[69] Vaughan DG, Marshall GJ, Connolley WM, Parkinson C, Mulvaney R, Hodgson DA, King JC, Pudsey CJ, Turner J (2003). Recent rapid regional climate warming on the Antarctic Peninsula. Clim Change.

[70] Wei T, Simko V, Levy M, Xie Y, Jin Y, Zemla J (2017). Package ‘corrplot’. Statistician.

[71] Wickham H. Data analysis. In: ggplot2. Use R!. Cham: Springer; 2016. pp. 189–201. 10.1007/978-3-319-24277-4_9.

[72] Yakimovich KM, Engstrom CB, Quarmby LM (2020). Alpine snow algae microbiome diversity in the Coast Range of British Columbia. Front Microbiol.

[73] Yang Z (1994). Maximum likelihood phylogenetic estimation from DNA sequences with variable rates over sites: approximate methods. J Mol Evol.

[74] Zhou J, Ning D (2017). Stochastic community assembly: does it matter in microbial ecology?. Microbiol Mol Biol Rev.

