# Supplementary material for: Antarctic snow algae: unraveling the processes underlying microbial community assembly during blooms formation
Source: Microbiome. 2023 Sep 5;11:200. doi: 10.1186/s40168-023-01643-6 (PMC10478455; doi:10.1186/s40168-023-01643-6)
Supplement: Supplementary file 2 — Additional file 1: Supplementary Fig. 1. Rarefaction curves of bacterial and eukaryotic (16S rRNA and 18S rRNA genes) ASVs. Supplementary Fig. 2. Shannon-Wiener diversity index and Richness (Chao 1) of bacterial and eukaryotic communities in snow samples collected at O’Higgins. Supplementary Fig. 3. Matrix for the correlations between the abundances of bacteria and eukaryotes. The figure illustrates positive (blue) and negative (red) significant correlations between taxa that most contributed to the assembly of the microbial community. The size of circles is proportional to the correlation value. Only significant correlations (p < 0.05) are illustrated. [file 40168_2023_1643_MOESM1_ESM.docx]

**Antarctic snow algae: unraveling the processes underlying microbial community assembly during blooms formation**

Daniela F. Soto,^1,2*^  Iván Gómez,^1,2^ and Pirjo Huovinen^1,2^

^1^Instituto de Ciencias Marinas y Limnológicas, Universidad Austral de Chile,Valdivia, Chile.

^2^ Research Centre on Dynamics of High Latitude Marine Ecosystems (IDEAL), Valdivia, Chile.

* **Corresponding author**:

Daniela F. Soto

Instituto de Ciencias Marinas y Limnológicas, Facultad de Ciencias, Edificio Emilio Pugin 1er Piso, Campus Isla Teja, Universidad Austral de Chile, Valdivia, Chile.

e-mail address: daniela.soto@uach.cl

phone/fax number: (56) 63 2221210 - 221455


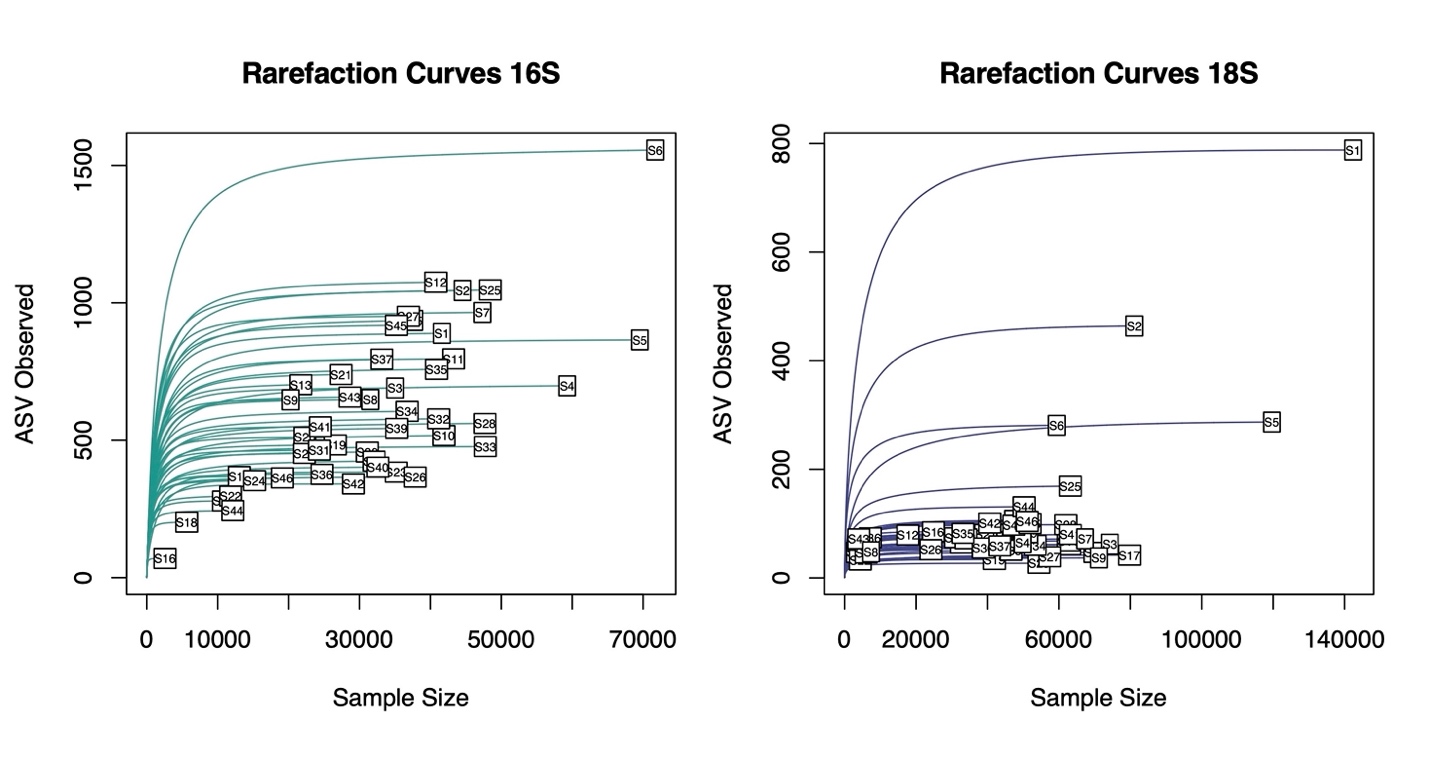


**Supplementary Fig. 1.** Rarefaction curves of bacterial and eukaryotic (16S rRNA and 18S rRNA genes) ASVs.


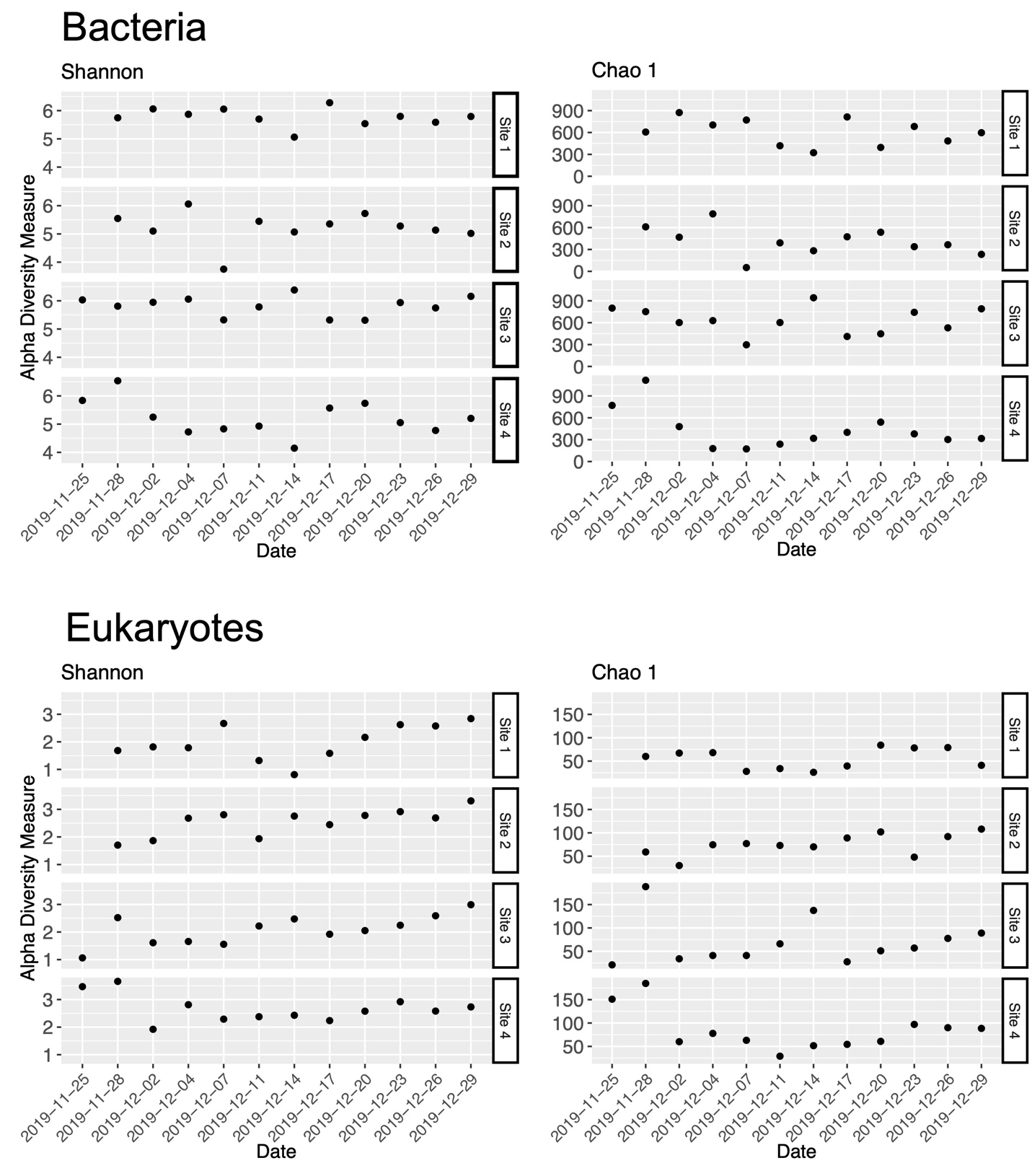


**Supplementary Fig. 2.** Shannon-Wiener diversity index and Richness (Chao 1) of bacterial and eukaryotic communities in snow samples collected at O’Higgins**.**


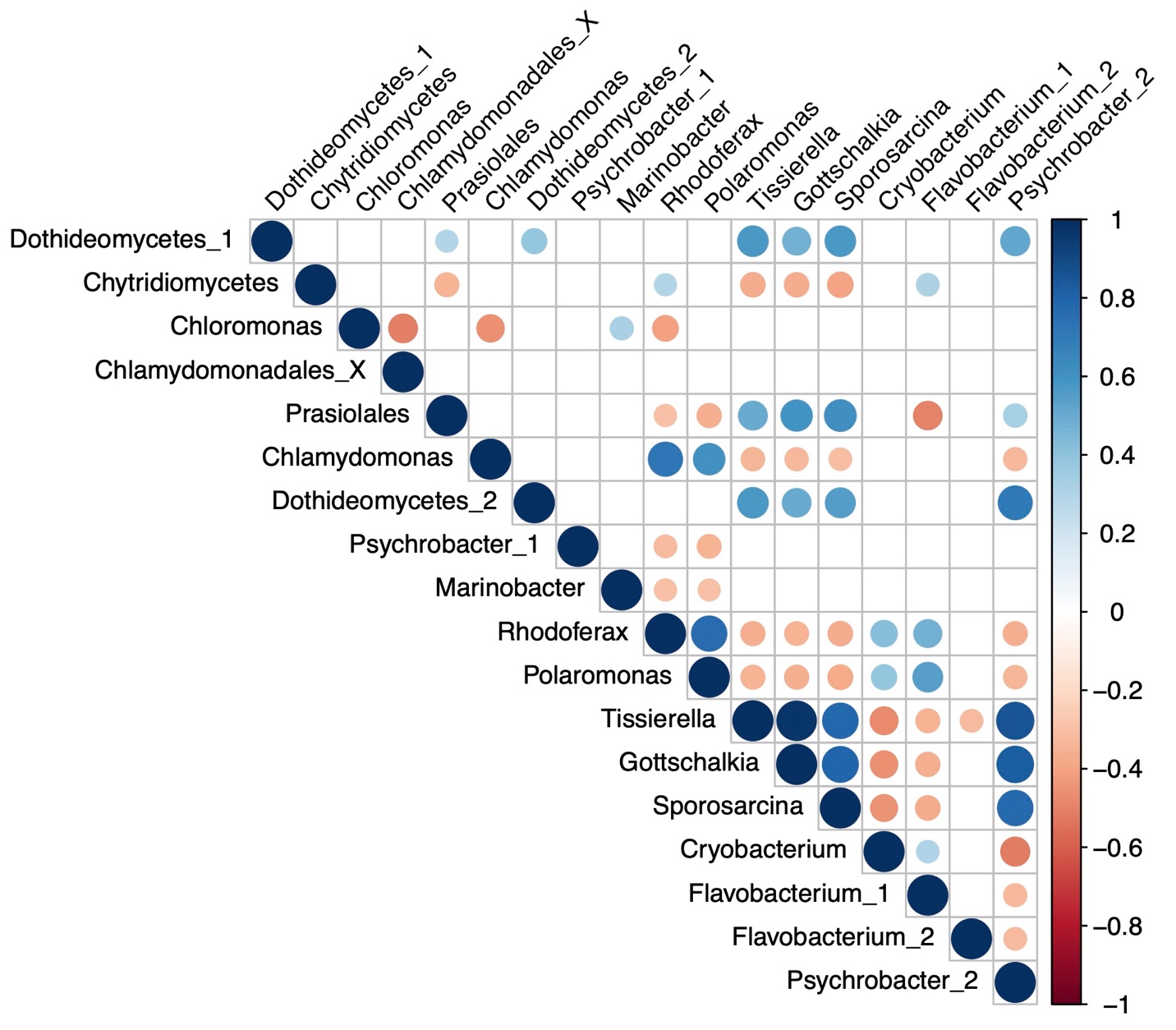


**Supplementary Fig. 3.** Matrix for the correlations between the abundances of bacteria and eukaryotes. The figure illustrates positive (blue) and negative (red) significant correlations between taxa that most contributed to the assembly of the microbial community. The size of circles is proportional to the correlation value. Only significant correlations (p < 0.05) are illustrated.
